# Crystal structures of 6a,6b,7,11a-tetra­hydro-6*H*,9*H*-spiro­[chromeno[3′,4′:3,4]pyrrolo­[1,2-*c*]thia­zole-11,3′-indoline]-2′,6-dione and 5′-methyl-6a,6b,7,11a-tetra­hydro-6*H*,9*H*-spiro­[chromeno[3′,4′:3,4]pyrrolo­[1,2-*c*]thia­zole-11,3′-indoline]-2′,6-dione

**DOI:** 10.1107/S2056989019000045

**Published:** 2019-01-22

**Authors:** S. Pangajavalli, R. Ranjithkumar, N. Srinivasan, S. Ramaswamy, S. Selvanayagam

**Affiliations:** a Department of Physics, Sri S. Ramasamy Naidu Memorial College, Sattur 626 203, India; b School of Chemistry, Madurai Kamaraj University, Madurai 625 021, India; c Department of Physics, Thiagarajar College, Madurai 625 009, India; d Department of Physics, N. M. S. S. Vellaichamy Nadar College, Madurai 625 019, India; e PG & Research Department of Physics, Government Arts College, Melur 625 106, India

**Keywords:** crystal structure, indole derivatives, pyrrolo, chromeno, spiro, thia­zole, N—H⋯π inter­actions, C—H⋯π inter­actions, hydrogen bonding

## Abstract

The title compounds, C_20_H_16_N_2_O_3_S, (I), and C_21_H_18_N_2_O_3_S, (II), differ by the presence of a methyl group in position 5 on the 1*H*-indole-2-one ring of compound (II). The two compounds have a structural overlap r.m.s. deviation of 0.48 Å. There is a significant difference in the conformation of the thia­zolidine ring: it has a twisted conformation on the fused N—C bond in (I), but an envelope conformation in compound (II) with the S atom as the flap. The planar pyrrolidine ring of the indole ring system is normal to the mean plane of the five-membered pyrrolidine ring of the pyrrolo­thia­zole unit in both compounds, with dihedral angles of 88.71 (9) and 84.59 (8)°. The pyran rings in both structures have envelope conformations with the methyl­ene C atom adjacent to the C=O group as the flap. In both compounds, there is a short intra­molecular C—H⋯O contact present. In the crystal of (I), mol­ecules are linked by C—H⋯O hydrogen bonds forming chains propagating along the *b*-axis direction. The chains are linked by N—H⋯π inter­actions, forming layers parallel to (10). In the crystal of (II), mol­ecules are linked by pairs of N—H⋯O hydrogen bonds, forming inversion dimers which are linked by C—H⋯O hydrogen bonds to form a three-dimensional structure.

## Abstract 2

The title compounds, (I) and (II), differ by the presence of a methyl group in position 5 on the 1*H*-indole-2-one ring of compound (II). There is also a significant difference in the conformation of the five-membered thia­zolidine ring in the two compounds.

## Chemical context   

Indole derivatives have been reported to exhibit a large number of biological activities, such as anti-inflammatory (Chen *et al.*, 2017 ▸), anti-fungal (Singh *et al.*, 2000 ▸), anti-hepatitis B virus (Chai *et al.*, 2006 ▸) and anti-HIV (Sriram *et al.*, 2006 ▸; Pandeya *et al.*, 2000 ▸). Indole analogues play a significant role in a diverse array of products, such as vitamin supplements, dyes, plastics, flavour enhancers, and in the agricultural and perfumery industries (Barden, 2011 ▸). In view of the importance of such compounds, we report herein on the synthesis and mol­ecular and crystal structures of the title compounds, 6a,6b,7,11a-tetra­hydro-6*H*,9*H*-spiro­[chromeno[3′,4′:3,4]pyrrolo [1,2-*c*]thia­zole-11,3′-indoline]-2′,6-dione (I) and 5′-methyl-6a,6b,7,11a-tetra­hydro-6*H*,9*H*-spiro­[chromeno[3′,4′:3,4]pyrrolo [1,2-*c*]thia­zole-11,3′-indoline]-2′,6-dione (II).
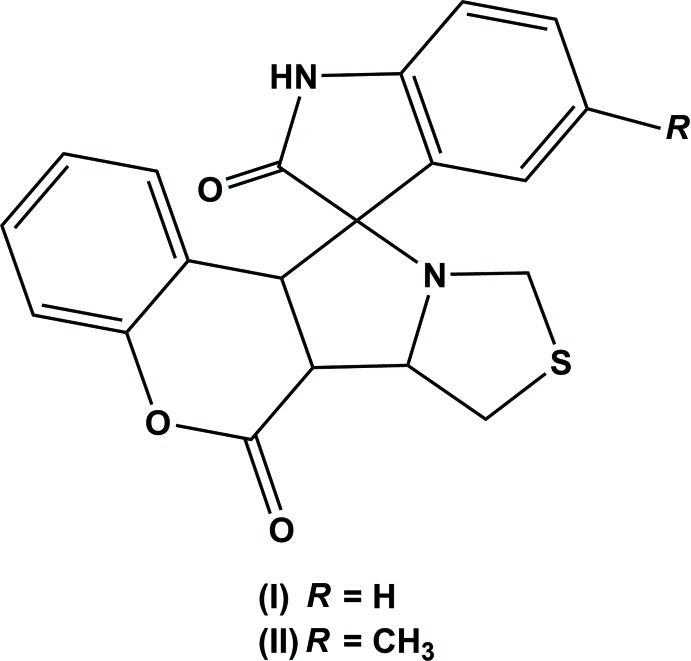


## Structural commentary   

**Figure 1:**
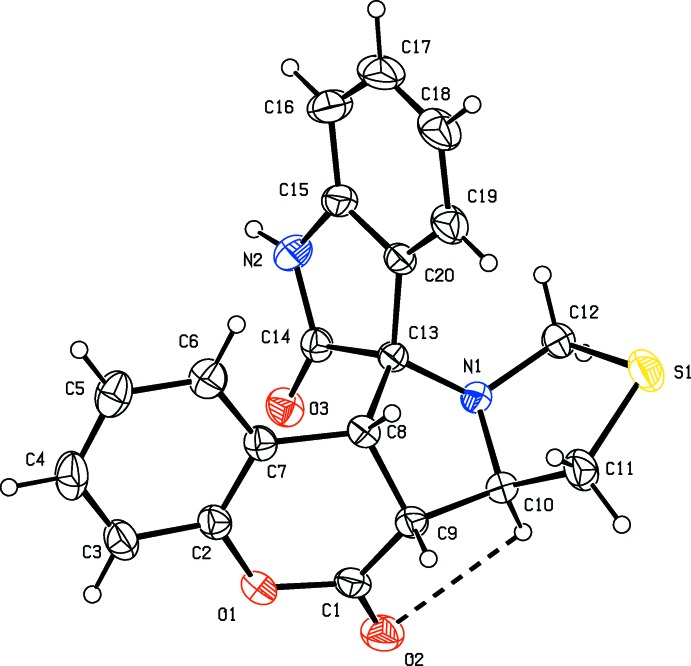
A view of the mol­ecular structure of compound (I), showing the atom labelling. Displacement ellipsoids are drawn at the 30% probability level. The intra­molecular C—H⋯O inter­action (Table 1 ▸) is shown as a dashed line.

**Figure 2:**
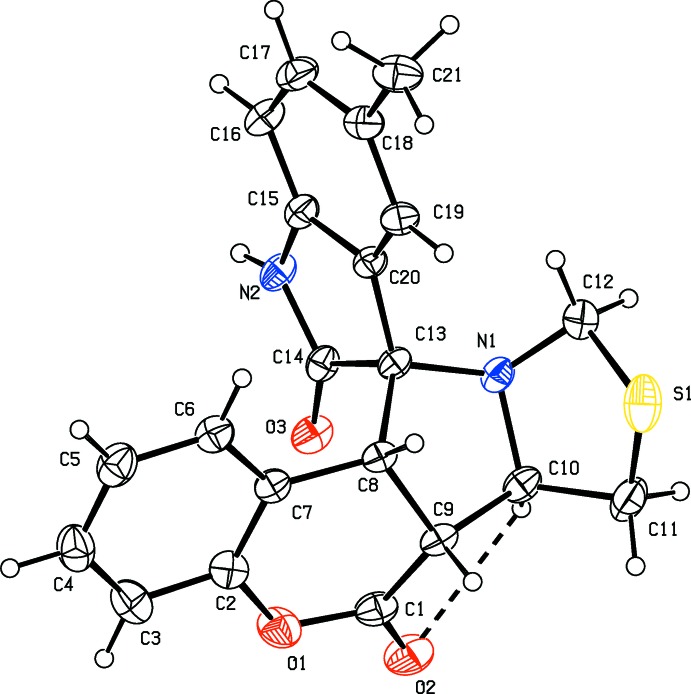
A view of the mol­ecular structure of compound (II), showing the atom labelling. Displacement ellipsoids are drawn at the 30% probability level. The intra­molecular C—H⋯O inter­action (Table 2 ▸) is shown as a dashed line.

**Figure 3:**
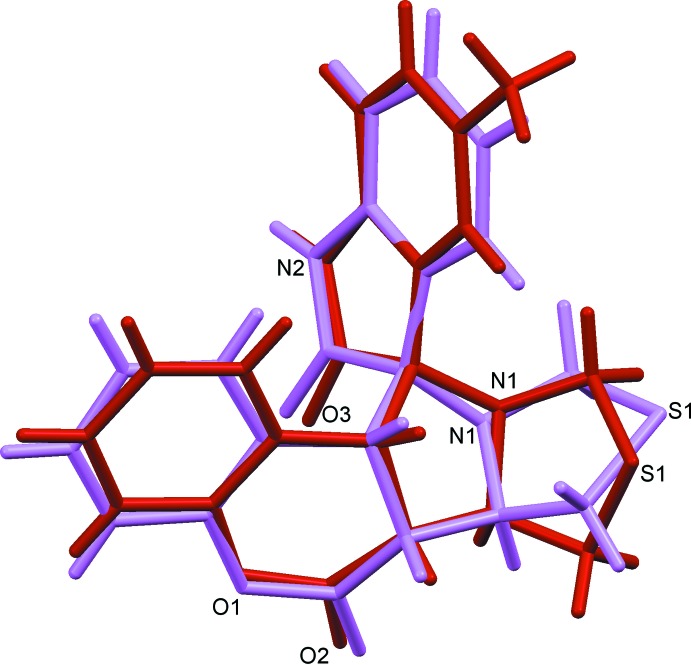
Structural overlay of compound (I) (purple) and compound (II) (red). The r.m.s. deviation is 0.48 Å (*Mercury*; Macrae *et al.*, 2008 ▸).

The mol­ecular structure of compound (I) is illustrated in Fig. 1 ▸, and for compound (II) in Fig. 2 ▸. The conformations of the two mol­ecules differ by an r.m.s. deviation of 0.48 Å, as shown in the structural overlap figure (Fig. 3 ▸). The mol­ecular structures of both compounds are influenced by a short intra­molecular C—H⋯O contact (Tables 1 ▸ and 2 ▸), which forms an *S*(5) ring motif (Figs. 1 ▸ and 2 ▸).

There is a significant difference in the conformation of the five-membered thia­zolidine ring in the two compounds. In compound (I), the thia­zolidine ring (S1/N1/C10–C12) adopts a twist conformation on the N1—C10 bond [Δ*C*_2_(S1) asymmetry parameter is 0.006 (1)]. In (II) this ring adopts an envelope conformation [puckering parameters *q*_2_ = 0.529 (2) Å and φ = 105.8 (1)°] with atom S1 as the flap, deviating by 0.896 (1) Å from the mean plane through the remaining four atoms.

In compound (I), the pyrrolidine ring (C8–C10/N1/C13) adopts an envelope conformation [puckering parameters *q*_2_ = 0.335 (2) Å and φ = 39.4 (1)°] with atom C9 as the flap, deviating by 0.518 (2) Å from the mean plane through the remaining four atoms. In (II) this ring adopts a twist conformation on the C8—C13 bond [Δ*C*_2_(C10) asymmetry parameter is 0.005 (1)].

The 2,3-di­hydro-1*H*-indol-2-one ring is planar in both compounds, with a maximum deviation of 0.054 (1) and 0.080 (1) Å from the mean plane for atom C14 in (I) and (II), respectively. Oxygen atom O3 of this ring deviates by 0.151 (1) and 0.185 (1) Å, respectively, from the above mean planes. The methyl atom C21 in (II) deviates by 0.056 (2) Å from the plane of the benzene ring to which it is attached.

The pyran rings (C1/O1/C2/C7–C9) in both structures have distorted sofa conformations, with Δ*C*_s_(C2) asymmetry parameters (Nardelli, 1983 ▸) of 0.005 (1) and 0.006 (1), respectively. Atom C9 deviates from the mean plane through the remaining five atoms (O1/C1/C2/C7/C8) of the pyran ring by 0.465 (2) Å in (I) and by 0.383 (2) Å in (II).

In both compounds, the planar pyrrolidine ring (N2/C13–C15/C20) of the indole ring system is normal to the mean plane of the pyrrolidine ring (N1/C8–C10/C13) of the pyrrolo­thia­zole unit, with a dihedral angle of 88.71 (9)° for (I) and 84.59 (8)° for (II). The mean plane of the pyrrolidine ring (N1/C8–C10/C13) is inclined to the mean plane of the thia­zolidine ring (S1/N1/C10–C12) by 64.39 (2)° in (I) and 79.51 (9)° in (II).

## Supra­molecular features   

**Figure 4:**
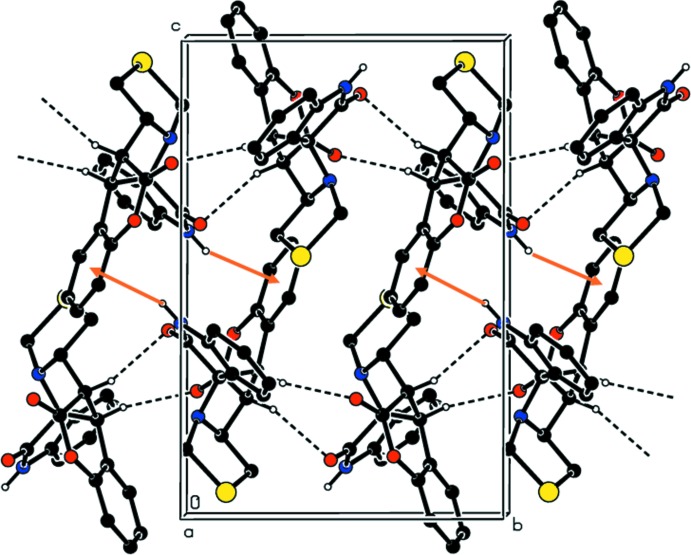
The crystal packing of compound (I) viewed along the *a* axis. The C—H⋯O hydrogen bonds (see Table 1 ▸) are shown as dashed lines, while the N—H⋯π inter­actions are shown as orange arrows. For clarity, H atoms not involved in these inter­actions have been omitted.

In the crystal of compound (I), mol­ecules associate *via* two C—H⋯O inter­molecular inter­actions (C8—H8⋯O2^ii^, C9—H9⋯O3^ii^, Table 1 ▸) forming chains propagating along [001]; see Fig. 4 ▸. In addition to this, inversion-related mol­ecules are linked to form dimers by N—H⋯π inter­actions; N2—H2⋯*Cg* ^i^, where *Cg* is the centroid of the benzene ring (C2–C7); see Fig. 4 ▸ and Table 1 ▸. The result of these inter­actions is the formation of layers lying parallel to the (10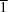) plane.

**Figure 5:**
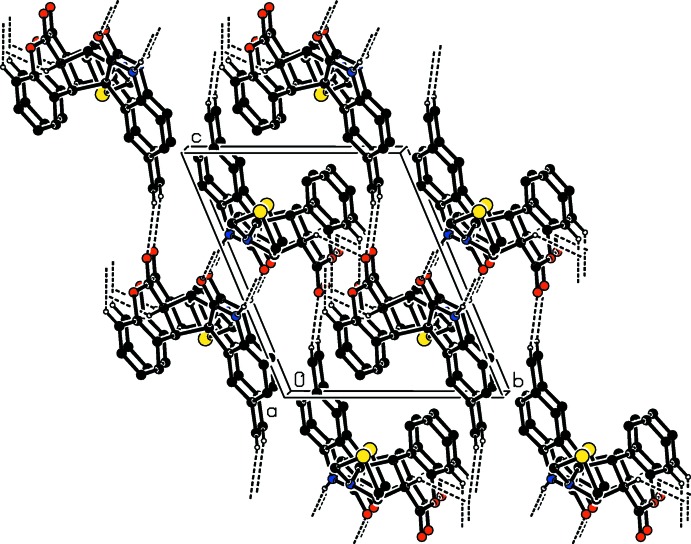
The crystal packing of compound (II) viewed along the *a* axis. The N—H⋯O and C—H⋯O hydrogen bonds (Table 2 ▸) are shown as dashed lines. For clarity, H atoms not involved in the hydrogen bonds have been omitted.

In the crystal of compound (II), mol­ecules are linked *via* pairs of N—H⋯O hydrogen bonds (N2—H2⋯O3^i^, Table 2 ▸), forming inversion dimers with an 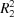
(8) ring motif (Fig. 5 ▸). There are two pairs of weak C—H⋯O inter­molecular inter­actions (C3—H3⋯O1^ii^, C9—H9⋯O2^iii^, Table 2 ▸) also forming inversion dimers and enclosing 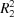
(8) ring motifs. These dimers are linked to form a helix along the *a*-axis direction. A further C—H⋯O hydrogen bond (C21—H21*C*⋯O2^iv^, Table 2 ▸) links the mol­ecules to form *C*(10) chains propagating along [010] in an anti-parallel manner. As a result of the various N—H⋯O and C—H⋯O hydrogen bonds, a three-dimensional structure is formed (Table 2 ▸ and Fig. 5 ▸)

## Database survey   

**Figure 6:**
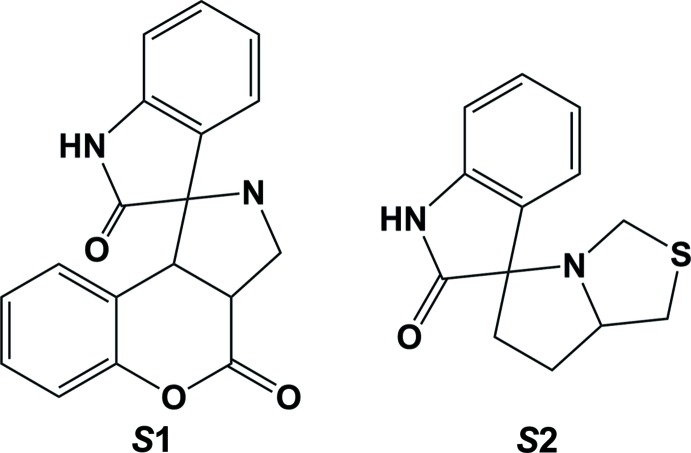
Partial structures for the CSD database searches.

A search of the Cambridge Structural Database (Version 5.39, last update August 2018; Groom *et al.*, 2016 ▸) for partial structure *S*1 (Fig. 6 ▸) gave three hits. Details are given in the supporting information (CSD search S1). They include: 2,4-di­chloro-5′-methyl-6a,6b,7,8,9,11a-hexa­hydro-6*H*-spiro[chromeno[3,4-*a*]pyrrolizine-11,3′-indole]-2′,6(1′*H*)-dione monohydrate (GUCGIN; Kanchithalaivan *et al.*, 2014*a* ▸), 3a-acetyl-2-methyl-2,3,3a,9b-tetra­hydro-4*H*-spiro­[chromeno[3,4-*c*]pyrrole-1,3′-indole]-2′,4(1′*H*)-dione (SUTLAV; Ghandi *et al.*, 2010 ▸), and 8-bromo-2-methyl-2,3,3a,9b-tetra­hydro-4*H*-spiro­[chromeno[3,4-*c*]pyrrole-1,3′-indole]-2′,4(1*′H*)-dione (SUTLEZ; Ghandi *et al.*, 2010 ▸). Here the dihedral angle between the planar pyrrolidine ring of the indole ring system and the mean plane of the pyrrolidine ring of the pyrrolo­thia­zole unit are 82.85, 87.66 and 86.60°, respectively, compared to 88.71 (9)° in (I) and 84.59 (8)° in (II).

A search for partial structure *S*2 (Fig. 6 ▸) gave 23 hits. Details are given in in the supporting information (CSD search S2). In these structures, the dihedral angle between the planar pyrrolidine ring of the indole ring system and the mean plane of the pyrrolidine ring of the pyrrolo­thia­zole unit varies from 77.60° in 1′-phenyl-6′-thia­cyclo­heptane-1-spiro-2′-perhydro­pyrrolizine-3′-spiro-3′′-indoline-2,2′′-dione (GITDOD; Sundaramoorthy *et al.*, 2008 ▸) to 89.72° in 3-hy­droxy-10,13-dimethyl-7′-(4-methyl­phen­yl)-1,3,4,5,6,7,7′,7a′,8,9,10,11,12,13,14,15-hexa­deca­hydro-1′*H*-di­spiro­[cyclo­penta­[*a*]phenanthrene-16,6′-pyrrolo­[1,2-*c*][1,3]thia­zole-5′,3′′-indole]-2′′,17(1′′*H*,2*H*)-dione (MUDLAA; Kanchithalaivan *et al.*, 2014*b* ▸). Only four of these compounds are mono­spiro, the others, like the two above, have a di­spiro arrangement. The four compounds are 7′-(2-chloro­phen­yl)-6′-(pyridin-2-ylcarbon­yl)-1′,6′,7′,7a′-tetra­hydro­spiro­[indole-3,5′-pyrrolo­[1,2-*c*][1,3]thiazol]-2(*1H*)-one ethanol solvate (GUCHET; Li *et al.*, 2014 ▸), ethyl 7′-(6-(benz­yloxy)-2,2-di­methyl­tetra­hydro­furo[2,3-*d*][1,3]dioxol-5-yl)-2-oxo-1,1′,2,6′,7′,7a′-hexa­hydro­spiro[indole-3,5′-pyrrolo­[1,2-*c*][1,3]thia­zole]-6′-carboxyl­ate (NUH­HIJ; Suhitha *et al.*, 2013 ▸), ethyl 2-oxo-7′-(2,2,7,7-tetra­methyl­tetra­hydro-3a*H*-bis­[1,3]dioxolo[4,5-*b*:4′,5′-*d*]pyran-5-yl)-1,1′,2,6′,7′,7a′-hexa­hydro­spiro­[indole-3,5′-pyrrolo­[1,2-*c*][1,3]thia­zole]-6′-carboxyl­ate monohydrate (SUWNEE; Prasanna *et al.*, 2010 ▸) and 6′-benzoyl-7′-(4-chloro­phen­yl)-3′-phenyl-1′,6′,7′,7a′-tetra­hydro­spiro­[indole-3,5′-pyrrolo­[1,2-*c*][1,3]thiazol]-2(1*H*)-one (XEVGIQ; Kumar *et al.*, 2013 ▸). Here the dihedral angles between the planar pyrrolidine ring of the indole ring system and the mean plane of the pyrrolidine ring of the pyrrolo­thia­zole unit are 79.94, 87.79, 84.78 and 81.44°, respectively, compared to 88.71 (9)° in (I) and 84.59 (8)° in (II).

## Synthesis and crystallization   

Compound (I): A flask containing salicyl­aldehyde (1 mmol) and 2,2-dimethyl-1,3-dioxane-4,6-dione (1 mmol) in water (7 ml) was placed at the maximum energy area in an ultrasonic cleaner and the surface of the reactants was placed slightly lower than the level of the water. The mixture was subjected to ultrasonic irradiation of low power at 323 K for *ca* 30 min. To this, a mixture of isatin (1 mmol) and 1,3-thia­zolane-4-carb­oxy­lic acid (1 mmol) dissolved in methanol (7 ml) was added. The irradiation was continued until the completion of the reaction (*ca* 50 min), during which time the product precipitated from the reaction mixture. It was then filtered and dried to obtain the pure product. The compound was further recrystallized from an ethanol–ethyl acetate mixture (1:1) to obtain colourless block-like crystals.

Compound (II): A flask containing salicyl­aldehyde (1 mmol) and 2,2-dimethyl-1,3-dioxane-4,6-dione (1 mmol) in water (7 ml) was placed at the maximum energy area in an ultrasonic cleaner and the surface of the reactants was placed slightly lower than the level of the water. The mixture was subjected to ultrasonic irradiation of low power at 323 K for about 30 min. To this, a mixture of 5-methyl­isatin (1 mmol) and 1,3-thia­zolane-4-carb­oxy­lic acid (1 mmol) dissolved in methanol (7 ml) was added. The irradiation was continued until the completion of the reaction (*ca* 45 min), during which time the product precipitated from the reaction mixture. It was then filtered and dried to obtain the pure product. The compound was further recrystallized from ethyl acetate to obtain colourless block-like crystals.

## Refinement   

Crystal data, data collection and structure refinement details are summarized in Table 3 ▸. For both compounds, the H atoms were placed in idealized positions and allowed to ride on their parent atoms: N—H = 0.86 Å and C—H = 0.93–0.97 Å, with *U*_iso_(H) = 1.5*U*_eq_(C-meth­yl) and 1.2*U*_eq_(N, C) for other H atoms.

## Supplementary Material

Crystal structure: contains datablock(s) I, II, global. DOI: 10.1107/S2056989019000045/su5467sup1.cif

Structure factors: contains datablock(s) I. DOI: 10.1107/S2056989019000045/su5467Isup2.hkl

Structure factors: contains datablock(s) II. DOI: 10.1107/S2056989019000045/su5467IIsup3.hkl

CSD search S1. DOI: 10.1107/S2056989019000045/su5467sup4.pdf

CSD search S2. DOI: 10.1107/S2056989019000045/su5467sup5.pdf

CCDC references: 1888373, 1888372

Additional supporting information:  crystallographic information; 3D view; checkCIF report

## Figures and Tables

**Table 1:** Hydrogen-bond geometry (Å, °) for (I) *Cg* is the centroid of the C2–C7 ring.

*D*—H⋯*A*	*D*—H	H⋯*A*	*D*⋯*A*	*D*—H⋯*A*
C10—H10⋯O2	0.98	2.50	2.902 (2)	104
N2—H2⋯*Cg* ^i^	0.86	2.57	3.799 (18)	157
C8—H8⋯O2^ii^	0.98	2.38	3.321 (2)	160
C9—H9⋯O3^ii^	0.98	2.44	3.376 (2)	159

Symmetry codes: (i) 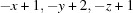; (ii) 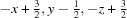.

**Table 2:** Hydrogen-bond geometry (Å, °) for (II)

*D*—H⋯*A*	*D*—H	H⋯*A*	*D*⋯*A*	*D*—H⋯*A*
C10—H10⋯O2	0.98	2.44	2.882 (2)	107
N2—H2⋯O3^i^	0.86	2.06	2.903 (2)	168
C3—H3⋯O1^ii^	0.93	2.55	3.302 (2)	139
C9—H9⋯O2^iii^	0.98	2.59	3.320 (2)	131
C21—H21*C*⋯O2^iv^	0.96	2.57	3.390 (2)	144

Symmetry codes: (i) 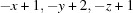; (ii) 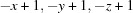; (iii) 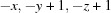; (iv) 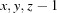.

**Table 3:** Experimental details

	(I)	(II)
Crystal data		
Chemical formula	C_20_H_16_N_2_O_3_S	C_21_H_18_N_2_O_3_S
*M*_r_	364.41	378.43
Crystal system, space group	Monoclinic, *P*2_1_/*n*	Triclinic, *P* 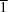
Temperature (K)	298	298
*a*, *b*, *c* (Å)	11.3058 (9), 10.0905 (8), 15.1957 (12)	8.3648 (5), 9.7648 (6), 11.9677 (7)
α, β, γ (°)	90, 101.072 (1), 90	112.622 (1), 99.388 (1), 91.885 (1)
*V* (Å^3^)	1701.3 (2)	885.31 (9)
*Z*	4	2
Radiation type	Mo *K*α	Mo *K*α
μ (mm^−1^)	0.21	0.21
Crystal size (mm)	0.21 × 0.18 × 0.16	0.22 × 0.19 × 0.17

Data collection		
Diffractometer	Bruker SMART APEX CCD area-detector	Bruker SMART APEX CCD area-detector
No. of measured, independent and observed [*I* > 2σ(*I*)] reflections	19453, 4146, 3646	10444, 4164, 3747
*R*_int_	0.023	0.016
(sin θ/λ)_max_ (Å^−1^)	0.668	0.666

Refinement		
*R*[*F* ^2^ > 2σ(*F* ^2^)], *wR*(*F* ^2^), *S*	0.052, 0.141, 1.02	0.048, 0.140, 1.05
No. of reflections	4146	4164
No. of parameters	235	245
No. of restraints	1	0
H-atom treatment	H-atom parameters constrained	H-atom parameters constrained
Δρ_max_, Δρ_min_ (e Å^−3^)	0.65, −0.37	0.67, −0.58

Computer programs: *SMART* and *SAINT* (Bruker, 2002 ▸), *SHELXS97* (Sheldrick, 2008 ▸), *SHELXL2018* (Sheldrick, 2015 ▸), *PLATON* (Spek, 2009 ▸), *Mercury* (Macrae *et al.*, 2008 ▸) and *publCIF* (Westrip, 2010 ▸).

## supplementary crystallographic information

### 6a,6b,7,11a-Tetrahydro-6H,9H-spiro[chromeno[3',4':3,4]pyrrolo[1,2-c]thiazole-11,3'-indoline]-2',6-dione (I) Crystal data

**Table:**

C_20_H_16_N_2_O_3_S	*F*(000) = 760
*M**_r_* = 364.41	*D*_x_ = 1.423 Mg m^−^^3^
Monoclinic, *P*2_1_/*n*	Mo *K*α radiation, λ = 0.71073 Å
*a* = 11.3058 (9) Å	Cell parameters from 11828 reflections
*b* = 10.0905 (8) Å	θ = 2.4–27.6°
*c* = 15.1957 (12) Å	µ = 0.21 mm^−^^1^
β = 101.072 (1)°	*T* = 298 K
*V* = 1701.3 (2) Å^3^	Block, colourless
*Z* = 4	0.21 × 0.18 × 0.16 mm

### 6a,6b,7,11a-Tetrahydro-6H,9H-spiro[chromeno[3',4':3,4]pyrrolo[1,2-c]thiazole-11,3'-indoline]-2',6-dione (I) Data collection

**Table:**

Bruker SMART APEX CCD area-detector diffractometer	*R*_int_ = 0.023
Radiation source: fine-focus sealed tube	θ_max_ = 28.4°, θ_min_ = 2.1°
ω and φ scans	*h* = −15→15
19453 measured reflections	*k* = −13→13
4146 independent reflections	*l* = −19→20
3646 reflections with *I* > 2σ(*I*)	

### 6a,6b,7,11a-Tetrahydro-6H,9H-spiro[chromeno[3',4':3,4]pyrrolo[1,2-c]thiazole-11,3'-indoline]-2',6-dione (I) Refinement

**Table:**

Refinement on *F*^2^	Primary atom site location: structure-invariant direct methods
Least-squares matrix: full	Secondary atom site location: difference Fourier map
*R*[*F*^2^ > 2σ(*F*^2^)] = 0.052	Hydrogen site location: inferred from neighbouring sites
*wR*(*F*^2^) = 0.141	H-atom parameters constrained
*S* = 1.02	*w* = 1/[σ^2^(*F*_o_^2^) + (0.0749*P*)^2^ + 0.6691*P*] where *P* = (*F*_o_^2^ + 2*F*_c_^2^)/3
4146 reflections	(Δ/σ)_max_ < 0.001
235 parameters	Δρ_max_ = 0.65 e Å^−^^3^
1 restraint	Δρ_min_ = −0.37 e Å^−^^3^

### 6a,6b,7,11a-Tetrahydro-6H,9H-spiro[chromeno[3',4':3,4]pyrrolo[1,2-c]thiazole-11,3'-indoline]-2',6-dione (I) Special details

**Table:**

Geometry. All esds (except the esd in the dihedral angle between two l.s. planes) are estimated using the full covariance matrix. The cell esds are taken into account individually in the estimation of esds in distances, angles and torsion angles; correlations between esds in cell parameters are only used when they are defined by crystal symmetry. An approximate (isotropic) treatment of cell esds is used for estimating esds involving l.s. planes.

### 6a,6b,7,11a-Tetrahydro-6H,9H-spiro[chromeno[3',4':3,4]pyrrolo[1,2-c]thiazole-11,3'-indoline]-2',6-dione (I) Fractional atomic coordinates and isotropic or equivalent isotropic displacement parameters (Å^2^)

**Table:**

	*x*	*y*	*z*	*U*_iso_*/*U*_eq_	
S1	0.57371 (5)	0.86992 (6)	0.95071 (3)	0.06229 (18)	
O1	0.84530 (11)	0.85243 (14)	0.62287 (8)	0.0483 (3)	
O2	0.91582 (12)	0.96789 (15)	0.74380 (10)	0.0580 (4)	
O3	0.63036 (12)	1.05167 (12)	0.61274 (9)	0.0491 (3)	
N1	0.61304 (11)	0.95792 (13)	0.79329 (8)	0.0363 (3)	
N2	0.42770 (14)	1.00915 (16)	0.59558 (11)	0.0510 (4)	
H2	0.401330	1.064837	0.553539	0.061*	
C1	0.84352 (14)	0.88731 (17)	0.70943 (12)	0.0414 (4)	
C2	0.75317 (15)	0.77756 (17)	0.57221 (11)	0.0413 (4)	
C3	0.77089 (19)	0.7398 (2)	0.48794 (13)	0.0545 (5)	
H3	0.842381	0.760585	0.469330	0.065*	
C4	0.6811 (2)	0.6711 (2)	0.43215 (13)	0.0619 (5)	
H4	0.692571	0.644363	0.375835	0.074*	
C5	0.5742 (2)	0.6417 (2)	0.45907 (13)	0.0585 (5)	
H5	0.512648	0.598674	0.420168	0.070*	
C6	0.55927 (18)	0.67675 (18)	0.54432 (13)	0.0496 (4)	
H6	0.487889	0.655046	0.562774	0.060*	
C7	0.64923 (15)	0.74391 (15)	0.60294 (11)	0.0383 (3)	
C8	0.63887 (13)	0.77552 (15)	0.69741 (10)	0.0351 (3)	
H8	0.608885	0.695840	0.722794	0.042*	
C9	0.75974 (14)	0.81137 (16)	0.75678 (10)	0.0382 (3)	
H9	0.799982	0.729271	0.780519	0.046*	
C10	0.72344 (14)	0.88742 (18)	0.83418 (10)	0.0417 (4)	
H10	0.786632	0.950538	0.859742	0.050*	
C11	0.6941 (2)	0.7942 (2)	0.90799 (13)	0.0611 (6)	
H11A	0.764283	0.783469	0.955452	0.073*	
H11B	0.669905	0.707638	0.883100	0.073*	
C12	0.54452 (15)	0.99367 (18)	0.86045 (11)	0.0433 (4)	
H12A	0.459238	0.995734	0.834284	0.052*	
H12B	0.568283	1.080964	0.884175	0.052*	
C13	0.55309 (13)	0.89158 (14)	0.70978 (10)	0.0325 (3)	
C14	0.54547 (15)	0.99491 (15)	0.63379 (10)	0.0375 (3)	
C15	0.35371 (15)	0.92197 (19)	0.63287 (12)	0.0465 (4)	
C16	0.23026 (18)	0.9034 (3)	0.60850 (16)	0.0679 (6)	
H16	0.183914	0.953834	0.563437	0.081*	
C17	0.17892 (19)	0.8076 (3)	0.65336 (18)	0.0767 (8)	
H17	0.096136	0.793850	0.638706	0.092*	
C18	0.2469 (2)	0.7318 (3)	0.71934 (17)	0.0718 (7)	
H18	0.209658	0.666702	0.747758	0.086*	
C19	0.37089 (18)	0.7509 (2)	0.74433 (13)	0.0547 (5)	
H19	0.417048	0.699062	0.788679	0.066*	
C20	0.42334 (14)	0.84931 (17)	0.70121 (10)	0.0391 (3)	

### 6a,6b,7,11a-Tetrahydro-6H,9H-spiro[chromeno[3',4':3,4]pyrrolo[1,2-c]thiazole-11,3'-indoline]-2',6-dione (I) Atomic displacement parameters (Å^2^)

**Table:**

	*U*^11^	*U*^22^	*U*^33^	*U*^12^	*U*^13^	*U*^23^
S1	0.0646 (3)	0.0793 (4)	0.0497 (3)	0.0140 (3)	0.0277 (2)	0.0133 (2)
O1	0.0390 (6)	0.0624 (8)	0.0475 (7)	0.0000 (5)	0.0180 (5)	0.0005 (6)
O2	0.0435 (7)	0.0635 (8)	0.0679 (9)	−0.0092 (6)	0.0132 (6)	−0.0084 (7)
O3	0.0530 (7)	0.0456 (7)	0.0512 (7)	−0.0028 (5)	0.0164 (5)	0.0105 (5)
N1	0.0341 (6)	0.0409 (7)	0.0333 (6)	0.0016 (5)	0.0047 (5)	−0.0035 (5)
N2	0.0463 (8)	0.0557 (9)	0.0473 (8)	0.0107 (7)	−0.0002 (6)	0.0104 (7)
C1	0.0321 (7)	0.0452 (9)	0.0481 (9)	0.0056 (6)	0.0107 (6)	0.0017 (7)
C2	0.0430 (8)	0.0418 (8)	0.0405 (8)	0.0097 (7)	0.0115 (7)	0.0029 (6)
C3	0.0605 (11)	0.0619 (11)	0.0458 (9)	0.0179 (9)	0.0218 (8)	0.0045 (8)
C4	0.0869 (15)	0.0603 (12)	0.0398 (9)	0.0177 (11)	0.0152 (9)	−0.0056 (8)
C5	0.0756 (14)	0.0496 (10)	0.0463 (10)	0.0016 (9)	0.0017 (9)	−0.0086 (8)
C6	0.0549 (10)	0.0423 (9)	0.0512 (10)	−0.0011 (8)	0.0090 (8)	−0.0056 (7)
C7	0.0443 (8)	0.0322 (7)	0.0394 (8)	0.0060 (6)	0.0105 (6)	−0.0008 (6)
C8	0.0366 (7)	0.0314 (7)	0.0388 (7)	0.0015 (6)	0.0111 (6)	0.0026 (6)
C9	0.0357 (7)	0.0418 (8)	0.0376 (7)	0.0066 (6)	0.0081 (6)	0.0046 (6)
C10	0.0334 (7)	0.0560 (10)	0.0352 (8)	0.0038 (7)	0.0053 (6)	−0.0002 (7)
C11	0.0646 (12)	0.0792 (14)	0.0438 (9)	0.0259 (11)	0.0210 (9)	0.0182 (9)
C12	0.0431 (8)	0.0475 (9)	0.0399 (8)	0.0052 (7)	0.0090 (6)	−0.0078 (7)
C13	0.0313 (7)	0.0341 (7)	0.0328 (7)	−0.0005 (5)	0.0079 (5)	−0.0005 (5)
C14	0.0419 (8)	0.0348 (7)	0.0362 (7)	0.0040 (6)	0.0087 (6)	−0.0006 (6)
C15	0.0356 (8)	0.0571 (10)	0.0457 (9)	0.0043 (7)	0.0054 (7)	−0.0124 (8)
C16	0.0381 (10)	0.0904 (16)	0.0697 (13)	0.0066 (10)	−0.0029 (9)	−0.0207 (12)
C17	0.0366 (10)	0.111 (2)	0.0841 (16)	−0.0196 (12)	0.0150 (10)	−0.0381 (15)
C18	0.0551 (12)	0.0933 (17)	0.0739 (14)	−0.0346 (12)	0.0293 (11)	−0.0258 (13)
C19	0.0510 (10)	0.0648 (12)	0.0519 (10)	−0.0176 (9)	0.0192 (8)	−0.0071 (9)
C20	0.0332 (7)	0.0479 (9)	0.0379 (8)	−0.0037 (6)	0.0108 (6)	−0.0097 (6)

### 6a,6b,7,11a-Tetrahydro-6H,9H-spiro[chromeno[3',4':3,4]pyrrolo[1,2-c]thiazole-11,3'-indoline]-2',6-dione (I) Geometric parameters (Å, º)

**Table:**

S1—C11	1.789 (2)	C8—C9	1.529 (2)
S1—C12	1.8373 (19)	C8—C13	1.555 (2)
O1—C1	1.366 (2)	C8—H8	0.9800
O1—C2	1.393 (2)	C9—C10	1.525 (2)
O2—C1	1.199 (2)	C9—H9	0.9800
O3—C14	1.212 (2)	C10—C11	1.548 (3)
N1—C12	1.441 (2)	C10—H10	0.9800
N1—C10	1.467 (2)	C11—H11A	0.9700
N1—C13	1.4791 (19)	C11—H11B	0.9700
N2—C14	1.354 (2)	C12—H12A	0.9700
N2—C15	1.406 (3)	C12—H12B	0.9700
N2—H2	0.8600	C13—C20	1.509 (2)
C1—C9	1.505 (2)	C13—C14	1.546 (2)
C2—C3	1.387 (2)	C15—C20	1.386 (3)
C2—C7	1.387 (2)	C15—C16	1.386 (3)
C3—C4	1.378 (3)	C16—C17	1.374 (4)
C3—H3	0.9300	C16—H16	0.9300
C4—C5	1.380 (3)	C17—C18	1.373 (4)
C4—H4	0.9300	C17—H17	0.9300
C5—C6	1.384 (3)	C18—C19	1.394 (3)
C5—H5	0.9300	C18—H18	0.9300
C6—C7	1.393 (2)	C19—C20	1.384 (3)
C6—H6	0.9300	C19—H19	0.9300
C7—C8	1.497 (2)		
			
C11—S1—C12	93.42 (8)	C9—C10—C11	112.32 (15)
C1—O1—C2	121.62 (13)	N1—C10—H10	110.4
C12—N1—C10	110.55 (12)	C9—C10—H10	110.4
C12—N1—C13	120.07 (13)	C11—C10—H10	110.4
C10—N1—C13	110.87 (12)	C10—C11—S1	106.68 (13)
C14—N2—C15	111.89 (14)	C10—C11—H11A	110.4
C14—N2—H2	124.1	S1—C11—H11A	110.4
C15—N2—H2	124.1	C10—C11—H11B	110.4
O2—C1—O1	117.34 (16)	S1—C11—H11B	110.4
O2—C1—C9	125.18 (17)	H11A—C11—H11B	108.6
O1—C1—C9	117.17 (15)	N1—C12—S1	108.34 (11)
C3—C2—C7	121.70 (17)	N1—C12—H12A	110.0
C3—C2—O1	115.71 (16)	S1—C12—H12A	110.0
C7—C2—O1	122.58 (14)	N1—C12—H12B	110.0
C4—C3—C2	119.08 (19)	S1—C12—H12B	110.0
C4—C3—H3	120.5	H12A—C12—H12B	108.4
C2—C3—H3	120.5	N1—C13—C20	118.66 (12)
C3—C4—C5	120.61 (18)	N1—C13—C14	106.63 (12)
C3—C4—H4	119.7	C20—C13—C14	102.26 (12)
C5—C4—H4	119.7	N1—C13—C8	104.53 (11)
C4—C5—C6	119.59 (19)	C20—C13—C8	113.28 (12)
C4—C5—H5	120.2	C14—C13—C8	111.37 (12)
C6—C5—H5	120.2	O3—C14—N2	126.85 (16)
C5—C6—C7	121.16 (19)	O3—C14—C13	125.62 (14)
C5—C6—H6	119.4	N2—C14—C13	107.52 (14)
C7—C6—H6	119.4	C20—C15—C16	121.8 (2)
C2—C7—C6	117.74 (16)	C20—C15—N2	109.67 (14)
C2—C7—C8	119.86 (15)	C16—C15—N2	128.57 (19)
C6—C7—C8	122.36 (15)	C17—C16—C15	117.5 (2)
C7—C8—C9	112.99 (13)	C17—C16—H16	121.2
C7—C8—C13	116.23 (12)	C15—C16—H16	121.2
C9—C8—C13	104.95 (12)	C18—C17—C16	121.6 (2)
C7—C8—H8	107.4	C18—C17—H17	119.2
C9—C8—H8	107.4	C16—C17—H17	119.2
C13—C8—H8	107.4	C17—C18—C19	121.0 (2)
C1—C9—C10	113.58 (14)	C17—C18—H18	119.5
C1—C9—C8	114.30 (13)	C19—C18—H18	119.5
C10—C9—C8	103.39 (12)	C20—C19—C18	118.0 (2)
C1—C9—H9	108.4	C20—C19—H19	121.0
C10—C9—H9	108.4	C18—C19—H19	121.0
C8—C9—H9	108.4	C19—C20—C15	120.05 (16)
N1—C10—C9	104.51 (12)	C19—C20—C13	131.26 (16)
N1—C10—C11	108.58 (13)	C15—C20—C13	108.54 (14)
			
C2—O1—C1—O2	−168.97 (15)	C11—S1—C12—N1	9.94 (14)
C2—O1—C1—C9	17.2 (2)	C12—N1—C13—C20	−6.6 (2)
C1—O1—C2—C3	−174.90 (15)	C10—N1—C13—C20	124.34 (15)
C1—O1—C2—C7	6.0 (2)	C12—N1—C13—C14	108.01 (15)
C7—C2—C3—C4	2.3 (3)	C10—N1—C13—C14	−121.09 (13)
O1—C2—C3—C4	−176.83 (16)	C12—N1—C13—C8	−133.94 (14)
C2—C3—C4—C5	0.9 (3)	C10—N1—C13—C8	−3.04 (15)
C3—C4—C5—C6	−2.7 (3)	C7—C8—C13—N1	−143.84 (13)
C4—C5—C6—C7	1.4 (3)	C9—C8—C13—N1	−18.23 (15)
C3—C2—C7—C6	−3.5 (3)	C7—C8—C13—C20	85.55 (16)
O1—C2—C7—C6	175.57 (15)	C9—C8—C13—C20	−148.84 (12)
C3—C2—C7—C8	174.22 (15)	C7—C8—C13—C14	−29.07 (18)
O1—C2—C7—C8	−6.7 (2)	C9—C8—C13—C14	96.54 (14)
C5—C6—C7—C2	1.6 (3)	C15—N2—C14—O3	176.23 (16)
C5—C6—C7—C8	−176.06 (17)	C15—N2—C14—C13	−3.47 (19)
C2—C7—C8—C9	−14.6 (2)	N1—C13—C14—O3	58.27 (19)
C6—C7—C8—C9	162.98 (15)	C20—C13—C14—O3	−176.48 (15)
C2—C7—C8—C13	106.78 (17)	C8—C13—C14—O3	−55.2 (2)
C6—C7—C8—C13	−75.59 (19)	N1—C13—C14—N2	−122.03 (14)
O2—C1—C9—C10	30.5 (2)	C20—C13—C14—N2	3.23 (16)
O1—C1—C9—C10	−156.16 (14)	C8—C13—C14—N2	124.51 (14)
O2—C1—C9—C8	148.82 (17)	C14—N2—C15—C20	2.3 (2)
O1—C1—C9—C8	−37.8 (2)	C14—N2—C15—C16	−176.71 (19)
C7—C8—C9—C1	35.47 (18)	C20—C15—C16—C17	−1.4 (3)
C13—C8—C9—C1	−92.13 (15)	N2—C15—C16—C17	177.50 (19)
C7—C8—C9—C10	159.44 (13)	C15—C16—C17—C18	−0.7 (3)
C13—C8—C9—C10	31.84 (15)	C16—C17—C18—C19	1.1 (4)
C12—N1—C10—C9	158.83 (13)	C17—C18—C19—C20	0.5 (3)
C13—N1—C10—C9	23.14 (17)	C18—C19—C20—C15	−2.5 (3)
C12—N1—C10—C11	38.78 (19)	C18—C19—C20—C13	−177.54 (17)
C13—N1—C10—C11	−96.91 (16)	C16—C15—C20—C19	3.0 (3)
C1—C9—C10—N1	90.83 (16)	N2—C15—C20—C19	−176.04 (16)
C8—C9—C10—N1	−33.61 (16)	C16—C15—C20—C13	179.08 (17)
C1—C9—C10—C11	−151.65 (15)	N2—C15—C20—C13	0.01 (18)
C8—C9—C10—C11	83.91 (16)	N1—C13—C20—C19	−69.5 (2)
N1—C10—C11—S1	−29.53 (19)	C14—C13—C20—C19	173.54 (17)
C9—C10—C11—S1	−144.59 (13)	C8—C13—C20—C19	53.6 (2)
C12—S1—C11—C10	11.05 (16)	N1—C13—C20—C15	115.00 (15)
C10—N1—C12—S1	−29.65 (16)	C14—C13—C20—C15	−1.91 (16)
C13—N1—C12—S1	101.39 (14)	C8—C13—C20—C15	−121.87 (14)

### 6a,6b,7,11a-Tetrahydro-6H,9H-spiro[chromeno[3',4':3,4]pyrrolo[1,2-c]thiazole-11,3'-indoline]-2',6-dione (I) Hydrogen-bond geometry (Å, º)

Cg is the centroid of the C2–C7 ring.

**Table:**

*D*—H···*A*	*D*—H	H···*A*	*D*···*A*	*D*—H···*A*
C10—H10···O2	0.98	2.50	2.902 (2)	104
N2—H2···*Cg*^i^	0.86	2.57	3.799 (18)	157
C8—H8···O2^ii^	0.98	2.38	3.321 (2)	160
C9—H9···O3^ii^	0.98	2.44	3.376 (2)	159

Symmetry codes: (i) −*x*+1, −*y*+2, −*z*+1; (ii) −*x*+3/2, *y*−1/2, −*z*+3/2.

### 5'-Methyl-6a,6b,7,11a-tetrahydro-6H,9H-spiro[chromeno[3',4':3,4]pyrrolo[1,2-c]thiazole-11,3'-indoline]-2',6-dione (II) Crystal data

**Table:**

C_21_H_18_N_2_O_3_S	*Z* = 2
*M**_r_* = 378.43	*F*(000) = 396
Triclinic, *P*1	*D*_x_ = 1.420 Mg m^−^^3^
*a* = 8.3648 (5) Å	Mo *K*α radiation, λ = 0.71073 Å
*b* = 9.7648 (6) Å	Cell parameters from 7888 reflections
*c* = 11.9677 (7) Å	θ = 1.9–27.2°
α = 112.622 (1)°	µ = 0.21 mm^−^^1^
β = 99.388 (1)°	*T* = 298 K
γ = 91.885 (1)°	Block, colourless
*V* = 885.31 (9) Å^3^	0.22 × 0.19 × 0.17 mm

### 5'-Methyl-6a,6b,7,11a-tetrahydro-6H,9H-spiro[chromeno[3',4':3,4]pyrrolo[1,2-c]thiazole-11,3'-indoline]-2',6-dione (II) Data collection

**Table:**

Bruker SMART APEX CCD area-detector diffractometer	*R*_int_ = 0.016
Radiation source: fine-focus sealed tube	θ_max_ = 28.3°, θ_min_ = 1.9°
ω and φ scans	*h* = −11→10
10444 measured reflections	*k* = −12→12
4164 independent reflections	*l* = −15→15
3747 reflections with *I* > 2σ(*I*)	

### 5'-Methyl-6a,6b,7,11a-tetrahydro-6H,9H-spiro[chromeno[3',4':3,4]pyrrolo[1,2-c]thiazole-11,3'-indoline]-2',6-dione (II) Refinement

**Table:**

Refinement on *F*^2^	0 restraints
Least-squares matrix: full	Hydrogen site location: mixed
*R*[*F*^2^ > 2σ(*F*^2^)] = 0.048	H-atom parameters constrained
*wR*(*F*^2^) = 0.140	*w* = 1/[σ^2^(*F*_o_^2^) + (0.0815*P*)^2^ + 0.2689*P*] where *P* = (*F*_o_^2^ + 2*F*_c_^2^)/3
*S* = 1.05	(Δ/σ)_max_ < 0.001
4164 reflections	Δρ_max_ = 0.67 e Å^−^^3^
245 parameters	Δρ_min_ = −0.58 e Å^−^^3^

### 5'-Methyl-6a,6b,7,11a-tetrahydro-6H,9H-spiro[chromeno[3',4':3,4]pyrrolo[1,2-c]thiazole-11,3'-indoline]-2',6-dione (II) Special details

**Table:**

Geometry. All esds (except the esd in the dihedral angle between two l.s. planes) are estimated using the full covariance matrix. The cell esds are taken into account individually in the estimation of esds in distances, angles and torsion angles; correlations between esds in cell parameters are only used when they are defined by crystal symmetry. An approximate (isotropic) treatment of cell esds is used for estimating esds involving l.s. planes.

### 5'-Methyl-6a,6b,7,11a-tetrahydro-6H,9H-spiro[chromeno[3',4':3,4]pyrrolo[1,2-c]thiazole-11,3'-indoline]-2',6-dione (II) Fractional atomic coordinates and isotropic or equivalent isotropic displacement parameters (Å^2^)

**Table:**

	*x*	*y*	*z*	*U*_iso_*/*U*_eq_	
S1	−0.29132 (5)	0.74000 (6)	0.23215 (5)	0.06325 (18)	
O1	0.28203 (16)	0.52624 (15)	0.43732 (11)	0.0523 (3)	
O2	0.13654 (18)	0.64554 (16)	0.57307 (11)	0.0577 (3)	
O3	0.33381 (14)	0.85985 (13)	0.48566 (10)	0.0430 (3)	
N1	0.00582 (14)	0.86415 (13)	0.35425 (11)	0.0349 (3)	
N2	0.38289 (15)	0.95200 (14)	0.34299 (12)	0.0385 (3)	
H2	0.470194	1.010156	0.384421	0.046*	
C1	0.1543 (2)	0.60502 (18)	0.46820 (14)	0.0429 (3)	
C2	0.3272 (2)	0.48986 (17)	0.32294 (14)	0.0412 (3)	
C3	0.4559 (2)	0.4032 (2)	0.30405 (18)	0.0530 (4)	
H3	0.504909	0.372078	0.364539	0.064*	
C4	0.5103 (2)	0.3637 (2)	0.19411 (19)	0.0543 (4)	
H4	0.597736	0.306867	0.180813	0.065*	
C5	0.4360 (2)	0.4081 (2)	0.10393 (18)	0.0516 (4)	
H5	0.472276	0.380135	0.029583	0.062*	
C6	0.3071 (2)	0.49431 (17)	0.12417 (15)	0.0415 (3)	
H6	0.257290	0.523540	0.062749	0.050*	
C7	0.25060 (17)	0.53824 (15)	0.23529 (13)	0.0344 (3)	
C8	0.10802 (17)	0.62702 (14)	0.25841 (12)	0.0304 (3)	
H8	0.022930	0.584116	0.184089	0.037*	
C9	0.03505 (18)	0.62333 (16)	0.36667 (12)	0.0342 (3)	
H9	−0.050188	0.539144	0.334102	0.041*	
C10	−0.04772 (19)	0.76820 (17)	0.41498 (13)	0.0384 (3)	
H10	−0.011738	0.818793	0.504508	0.046*	
C11	−0.2345 (2)	0.7416 (2)	0.3835 (2)	0.0562 (5)	
H11A	−0.281609	0.820745	0.442410	0.067*	
H11B	−0.271795	0.647102	0.384579	0.067*	
C12	−0.1351 (2)	0.8969 (2)	0.28430 (17)	0.0490 (4)	
H12A	−0.105957	0.911235	0.214080	0.059*	
H12B	−0.175301	0.987402	0.335626	0.059*	
C13	0.13847 (16)	0.79580 (15)	0.28968 (12)	0.0310 (3)	
C14	0.29735 (17)	0.87016 (15)	0.38586 (13)	0.0338 (3)	
C15	0.31155 (17)	0.93035 (16)	0.22215 (14)	0.0355 (3)	
C16	0.3694 (2)	0.98465 (19)	0.14429 (16)	0.0463 (4)	
H16	0.466289	1.047091	0.169880	0.056*	
C17	0.2784 (2)	0.94312 (19)	0.02629 (16)	0.0470 (4)	
H17	0.315011	0.980334	−0.026878	0.056*	
C18	0.1343 (2)	0.84777 (16)	−0.01552 (14)	0.0398 (3)	
C19	0.07923 (19)	0.79402 (15)	0.06572 (13)	0.0365 (3)	
H19	−0.015862	0.729181	0.039691	0.044*	
C20	0.16607 (17)	0.83718 (14)	0.18489 (13)	0.0321 (3)	
C21	0.0385 (3)	0.8044 (2)	−0.14405 (15)	0.0522 (4)	
H21A	−0.023462	0.884117	−0.147536	0.078*	
H21B	−0.034059	0.716052	−0.165745	0.078*	
H21C	0.111978	0.785222	−0.200908	0.078*	

### 5'-Methyl-6a,6b,7,11a-tetrahydro-6H,9H-spiro[chromeno[3',4':3,4]pyrrolo[1,2-c]thiazole-11,3'-indoline]-2',6-dione (II) Atomic displacement parameters (Å^2^)

**Table:**

	*U*^11^	*U*^22^	*U*^33^	*U*^12^	*U*^13^	*U*^23^
S1	0.0352 (2)	0.0722 (3)	0.0593 (3)	0.0004 (2)	−0.00072 (19)	0.0054 (2)
O1	0.0604 (8)	0.0633 (8)	0.0366 (6)	0.0138 (6)	0.0050 (5)	0.0245 (6)
O2	0.0719 (9)	0.0725 (9)	0.0334 (6)	0.0018 (7)	0.0122 (6)	0.0255 (6)
O3	0.0408 (6)	0.0495 (6)	0.0329 (5)	−0.0105 (5)	−0.0009 (4)	0.0144 (5)
N1	0.0321 (6)	0.0334 (6)	0.0370 (6)	−0.0014 (4)	0.0093 (5)	0.0109 (5)
N2	0.0311 (6)	0.0413 (7)	0.0378 (6)	−0.0093 (5)	0.0043 (5)	0.0120 (5)
C1	0.0512 (9)	0.0462 (8)	0.0328 (7)	−0.0035 (7)	0.0057 (6)	0.0188 (6)
C2	0.0433 (8)	0.0394 (7)	0.0361 (7)	0.0003 (6)	0.0026 (6)	0.0120 (6)
C3	0.0510 (10)	0.0467 (9)	0.0545 (10)	0.0085 (7)	−0.0017 (8)	0.0171 (8)
C4	0.0463 (9)	0.0441 (9)	0.0646 (11)	0.0098 (7)	0.0112 (8)	0.0124 (8)
C5	0.0528 (10)	0.0450 (9)	0.0531 (10)	0.0035 (7)	0.0211 (8)	0.0110 (7)
C6	0.0462 (8)	0.0383 (7)	0.0386 (8)	0.0020 (6)	0.0115 (6)	0.0126 (6)
C7	0.0361 (7)	0.0298 (6)	0.0330 (7)	−0.0029 (5)	0.0047 (5)	0.0091 (5)
C8	0.0335 (6)	0.0303 (6)	0.0254 (6)	−0.0037 (5)	0.0041 (5)	0.0100 (5)
C9	0.0385 (7)	0.0351 (7)	0.0280 (6)	−0.0053 (5)	0.0070 (5)	0.0118 (5)
C10	0.0426 (8)	0.0384 (7)	0.0312 (7)	−0.0027 (6)	0.0121 (6)	0.0093 (6)
C11	0.0434 (9)	0.0535 (10)	0.0752 (13)	0.0006 (7)	0.0276 (9)	0.0233 (9)
C12	0.0419 (8)	0.0568 (10)	0.0570 (10)	0.0120 (7)	0.0164 (7)	0.0286 (8)
C13	0.0302 (6)	0.0305 (6)	0.0295 (6)	−0.0040 (5)	0.0048 (5)	0.0097 (5)
C14	0.0306 (6)	0.0315 (6)	0.0338 (7)	−0.0028 (5)	0.0062 (5)	0.0073 (5)
C15	0.0343 (7)	0.0340 (7)	0.0376 (7)	−0.0003 (5)	0.0100 (6)	0.0127 (6)
C16	0.0438 (8)	0.0475 (9)	0.0511 (9)	−0.0062 (7)	0.0158 (7)	0.0214 (7)
C17	0.0567 (10)	0.0471 (9)	0.0470 (9)	0.0038 (7)	0.0235 (8)	0.0240 (7)
C18	0.0538 (9)	0.0330 (7)	0.0344 (7)	0.0076 (6)	0.0137 (6)	0.0132 (6)
C19	0.0425 (8)	0.0311 (7)	0.0342 (7)	−0.0015 (5)	0.0061 (6)	0.0118 (5)
C20	0.0338 (7)	0.0289 (6)	0.0335 (7)	−0.0006 (5)	0.0081 (5)	0.0117 (5)
C21	0.0774 (12)	0.0452 (9)	0.0342 (8)	0.0052 (8)	0.0107 (8)	0.0159 (7)

### 5'-Methyl-6a,6b,7,11a-tetrahydro-6H,9H-spiro[chromeno[3',4':3,4]pyrrolo[1,2-c]thiazole-11,3'-indoline]-2',6-dione (II) Geometric parameters (Å, º)

**Table:**

S1—C11	1.790 (2)	C8—C13	1.5453 (18)
S1—C12	1.8191 (19)	C8—H8	0.9800
O1—C1	1.355 (2)	C9—C10	1.543 (2)
O1—C2	1.395 (2)	C9—H9	0.9800
O2—C1	1.1985 (19)	C10—C11	1.535 (2)
O3—C14	1.2270 (18)	C10—H10	0.9800
N1—C12	1.450 (2)	C11—H11A	0.9700
N1—C13	1.4851 (17)	C11—H11B	0.9700
N1—C10	1.4862 (18)	C12—H12A	0.9700
N2—C14	1.3458 (18)	C12—H12B	0.9700
N2—C15	1.4026 (19)	C13—C20	1.5058 (18)
N2—H2	0.8600	C13—C14	1.5534 (18)
C1—C9	1.512 (2)	C15—C16	1.376 (2)
C2—C7	1.384 (2)	C15—C20	1.3948 (19)
C2—C3	1.385 (2)	C16—C17	1.388 (2)
C3—C4	1.380 (3)	C16—H16	0.9300
C3—H3	0.9300	C17—C18	1.393 (2)
C4—C5	1.377 (3)	C17—H17	0.9300
C4—H4	0.9300	C18—C19	1.398 (2)
C5—C6	1.385 (2)	C18—C21	1.504 (2)
C5—H5	0.9300	C19—C20	1.385 (2)
C6—C7	1.399 (2)	C19—H19	0.9300
C6—H6	0.9300	C21—H21A	0.9600
C7—C8	1.497 (2)	C21—H21B	0.9600
C8—C9	1.5314 (18)	C21—H21C	0.9600
			
C11—S1—C12	85.68 (8)	C9—C10—H10	109.6
C1—O1—C2	122.33 (12)	C10—C11—S1	105.85 (11)
C12—N1—C13	118.56 (12)	C10—C11—H11A	110.6
C12—N1—C10	109.63 (12)	S1—C11—H11A	110.6
C13—N1—C10	108.19 (11)	C10—C11—H11B	110.6
C14—N2—C15	111.38 (11)	S1—C11—H11B	110.6
C14—N2—H2	124.3	H11A—C11—H11B	108.7
C15—N2—H2	124.3	N1—C12—S1	107.95 (11)
O2—C1—O1	117.29 (15)	N1—C12—H12A	110.1
O2—C1—C9	124.23 (17)	S1—C12—H12A	110.1
O1—C1—C9	118.21 (13)	N1—C12—H12B	110.1
C7—C2—C3	122.38 (16)	S1—C12—H12B	110.1
C7—C2—O1	122.58 (15)	H12A—C12—H12B	108.4
C3—C2—O1	115.03 (15)	N1—C13—C20	116.57 (11)
C4—C3—C2	118.98 (17)	N1—C13—C8	105.43 (10)
C4—C3—H3	120.5	C20—C13—C8	115.84 (11)
C2—C3—H3	120.5	N1—C13—C14	104.40 (10)
C5—C4—C3	120.40 (17)	C20—C13—C14	101.50 (11)
C5—C4—H4	119.8	C8—C13—C14	112.66 (11)
C3—C4—H4	119.8	O3—C14—N2	126.47 (13)
C4—C5—C6	119.87 (17)	O3—C14—C13	125.21 (12)
C4—C5—H5	120.1	N2—C14—C13	108.26 (12)
C6—C5—H5	120.1	C16—C15—C20	121.57 (14)
C5—C6—C7	121.20 (16)	C16—C15—N2	128.55 (14)
C5—C6—H6	119.4	C20—C15—N2	109.85 (12)
C7—C6—H6	119.4	C15—C16—C17	117.78 (15)
C2—C7—C6	117.15 (14)	C15—C16—H16	121.1
C2—C7—C8	120.27 (13)	C17—C16—H16	121.1
C6—C7—C8	122.51 (13)	C16—C17—C18	122.49 (14)
C7—C8—C9	113.73 (11)	C16—C17—H17	118.8
C7—C8—C13	116.91 (11)	C18—C17—H17	118.8
C9—C8—C13	102.92 (11)	C17—C18—C19	118.28 (14)
C7—C8—H8	107.6	C17—C18—C21	121.29 (14)
C9—C8—H8	107.6	C19—C18—C21	120.43 (15)
C13—C8—H8	107.6	C20—C19—C18	120.12 (14)
C1—C9—C8	115.20 (12)	C20—C19—H19	119.9
C1—C9—C10	111.88 (12)	C18—C19—H19	119.9
C8—C9—C10	106.10 (11)	C19—C20—C15	119.71 (13)
C1—C9—H9	107.8	C19—C20—C13	131.67 (12)
C8—C9—H9	107.8	C15—C20—C13	108.54 (12)
C10—C9—H9	107.8	C18—C21—H21A	109.5
N1—C10—C11	108.17 (13)	C18—C21—H21B	109.5
N1—C10—C9	106.68 (11)	H21A—C21—H21B	109.5
C11—C10—C9	113.15 (13)	C18—C21—H21C	109.5
N1—C10—H10	109.6	H21A—C21—H21C	109.5
C11—C10—H10	109.6	H21B—C21—H21C	109.5
			
C2—O1—C1—O2	−171.58 (15)	C12—N1—C13—C20	31.56 (17)
C2—O1—C1—C9	14.1 (2)	C10—N1—C13—C20	157.10 (12)
C1—O1—C2—C7	3.6 (2)	C12—N1—C13—C8	−98.51 (14)
C1—O1—C2—C3	−177.22 (15)	C10—N1—C13—C8	27.03 (14)
C7—C2—C3—C4	0.2 (3)	C12—N1—C13—C14	142.58 (13)
O1—C2—C3—C4	−179.05 (16)	C10—N1—C13—C14	−91.88 (12)
C2—C3—C4—C5	−1.0 (3)	C7—C8—C13—N1	−158.43 (11)
C3—C4—C5—C6	0.8 (3)	C9—C8—C13—N1	−33.02 (13)
C4—C5—C6—C7	0.2 (3)	C7—C8—C13—C20	71.07 (15)
C3—C2—C7—C6	0.8 (2)	C9—C8—C13—C20	−163.51 (12)
O1—C2—C7—C6	179.97 (14)	C7—C8—C13—C14	−45.18 (16)
C3—C2—C7—C8	177.75 (15)	C9—C8—C13—C14	80.23 (13)
O1—C2—C7—C8	−3.1 (2)	C15—N2—C14—O3	176.06 (14)
C5—C6—C7—C2	−1.0 (2)	C15—N2—C14—C13	−6.65 (16)
C5—C6—C7—C8	−177.86 (14)	N1—C13—C14—O3	62.34 (17)
C2—C7—C8—C9	−14.12 (18)	C20—C13—C14—O3	−176.09 (14)
C6—C7—C8—C9	162.65 (13)	C8—C13—C14—O3	−51.55 (18)
C2—C7—C8—C13	105.69 (15)	N1—C13—C14—N2	−115.00 (12)
C6—C7—C8—C13	−77.55 (17)	C20—C13—C14—N2	6.58 (14)
O2—C1—C9—C8	155.28 (16)	C8—C13—C14—N2	131.12 (12)
O1—C1—C9—C8	−30.88 (19)	C14—N2—C15—C16	−174.38 (16)
O2—C1—C9—C10	34.0 (2)	C14—N2—C15—C20	3.89 (17)
O1—C1—C9—C10	−152.16 (13)	C20—C15—C16—C17	−0.3 (2)
C7—C8—C9—C1	29.87 (17)	N2—C15—C16—C17	177.81 (15)
C13—C8—C9—C1	−97.58 (14)	C15—C16—C17—C18	−1.1 (3)
C7—C8—C9—C10	154.24 (11)	C16—C17—C18—C19	0.8 (2)
C13—C8—C9—C10	26.79 (14)	C16—C17—C18—C21	−179.81 (16)
C12—N1—C10—C11	−1.27 (17)	C17—C18—C19—C20	0.8 (2)
C13—N1—C10—C11	−131.91 (13)	C21—C18—C19—C20	−178.52 (14)
C12—N1—C10—C9	120.75 (13)	C18—C19—C20—C15	−2.2 (2)
C13—N1—C10—C9	−9.89 (14)	C18—C19—C20—C13	−178.66 (14)
C1—C9—C10—N1	115.18 (13)	C16—C15—C20—C19	1.9 (2)
C8—C9—C10—N1	−11.22 (15)	N2—C15—C20—C19	−176.49 (13)
C1—C9—C10—C11	−125.99 (15)	C16—C15—C20—C13	179.15 (14)
C8—C9—C10—C11	107.60 (14)	N2—C15—C20—C13	0.73 (16)
N1—C10—C11—S1	32.33 (15)	N1—C13—C20—C19	−74.82 (19)
C9—C10—C11—S1	−85.63 (14)	C8—C13—C20—C19	50.1 (2)
C12—S1—C11—C10	−41.61 (12)	C14—C13—C20—C19	172.50 (15)
C13—N1—C12—S1	94.75 (13)	N1—C13—C20—C15	108.41 (13)
C10—N1—C12—S1	−30.09 (15)	C8—C13—C20—C15	−126.64 (13)
C11—S1—C12—N1	42.32 (12)	C14—C13—C20—C15	−4.27 (14)

### 5'-Methyl-6a,6b,7,11a-tetrahydro-6H,9H-spiro[chromeno[3',4':3,4]pyrrolo[1,2-c]thiazole-11,3'-indoline]-2',6-dione (II) Hydrogen-bond geometry (Å, º)

**Table:**

*D*—H···*A*	*D*—H	H···*A*	*D*···*A*	*D*—H···*A*
C10—H10···O2	0.98	2.44	2.882 (2)	107
N2—H2···O3^i^	0.86	2.06	2.903 (2)	168
C3—H3···O1^ii^	0.93	2.55	3.302 (2)	139
C9—H9···O2^iii^	0.98	2.59	3.320 (2)	131
C21—H21*C*···O2^iv^	0.96	2.57	3.390 (2)	144

Symmetry codes: (i) −*x*+1, −*y*+2, −*z*+1; (ii) −*x*+1, −*y*+1, −*z*+1; (iii) −*x*, −*y*+1, −*z*+1; (iv) *x*, *y*, *z*−1.
