# Supplementary material for: Crystal structures of 6a,6b,7,11a-tetra­hydro-6H,9H-spiro­[chromeno[3′,4′:3,4]pyrrolo­[1,2-c]thia­zole-11,3′-indoline]-2′,6-dione and 5′-methyl-6a,6b,7,11a-tetra­hydro-6H,9H-spiro­[chromeno[3′,4′:3,4]pyrrolo­[1,2-c]thia­zole-11,3′-indoline]-2′,6-dione
Source: Acta Crystallogr E Crystallogr Commun. 2019 Jan 22;75(Pt 2):246–50. doi: 10.1107/S2056989019000045 (PMC6362654; doi:10.1107/S2056989019000045)

Search Overview

Search:	search2
Date/Time done:	Sat Dec 29 16:55:50 2018
Database(s):	CSD version 5.39 updates (Nov 2017)<br>CSD version 5.39 (November 2017)<br>CSD version 5.39 (November 2017)<br>CSD version 5.39 updates (Feb 2018)<br>CSD version 5.39 updates (May 2018)<br>CSD version 5.39 updates (Aug 2018)
Restriction Info:	No refcode restrictions applied
Filters:	None
Percentage Completed:	100%
Number of Hits:	3

Single query used. Search found structures that:

match
Query 1

Query 1

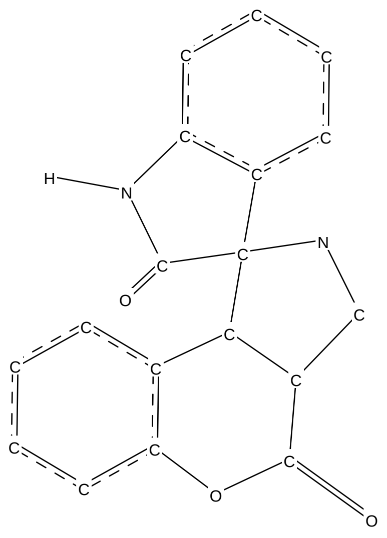

Search: search2 (Sat Dec 29 16:55:50 2018): Hits 1-2

GUCGIW

Reference: S.Kanchithalaivan, R.V.Sumesh, R.R.Kumar (2014) *ACS Comb. Sci.* ,**16,**566

Formula: $C_{22}\ H_{18}\ Cl_2\ N_2\ O_3,H_2\ O_1$

Compound Name: 2,4-Dichloro-5'-methyl-6a,6b,7,8,9,11a-hexahydro-6H-spiro[chromeno[3,4-a]pyrrolizine-11,3'-indole]-2',6(1'H)-dione monohydrate

Space Group:	P21/c	***Cell:***	***a***	12.301(0)	***b***	18.849(0)	***c***	10.439(0)
Space Group No.:	14	***(Å,°)***	α	90.00	β	98.50(0)	γ	90.00

R-Factor (%): 8.13 ***Temperature(K):*** 293 ***Density(g/cm^3):*** 1.241

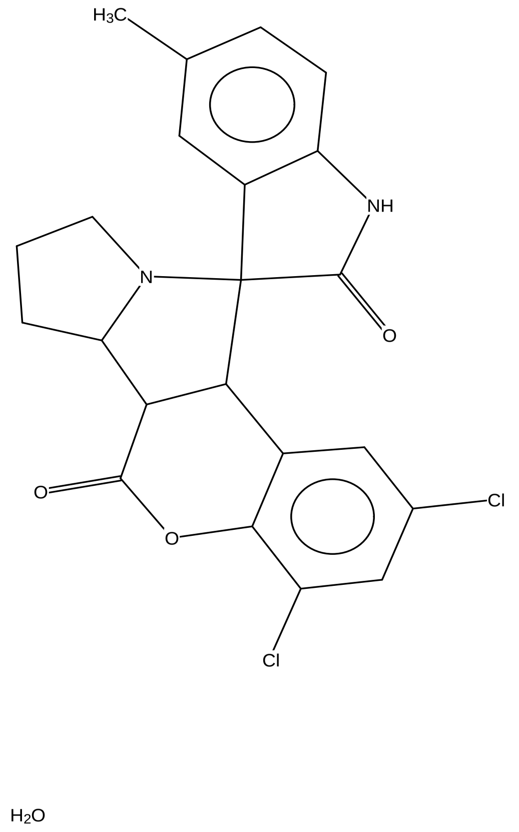

Parameters

Fragment 1

ANG1 (A) 82.853

SUTLAV

Reference: M.Ghandi, A.Taheri, A.Abbasi (2010) *Tetrahedron* ,**66,** 6744

Formula: $C_{21}\ H_{18}\ N_2\ O_4$

Compound Name: 3a-Acetyl-2-methyl-2,3,3a,9b-tetrahydro-4H-spiro[chromeno[3,4-c]pyrrole-1,3'-indole]-2',4(1'H)-dione

Space Group:	P-1	***Cell:***	***a***	8.542(1)	***b***	8.660(1)	***c***	12.791(3)
Space Group No.:	2	***(Å,°)***	α	107.43(3)	β	91.16(1)	γ	101.93(2)

R-Factor (%): 6.52 ***Temperature(K):*** 295 ***Density(g/cm^3):*** 1.368

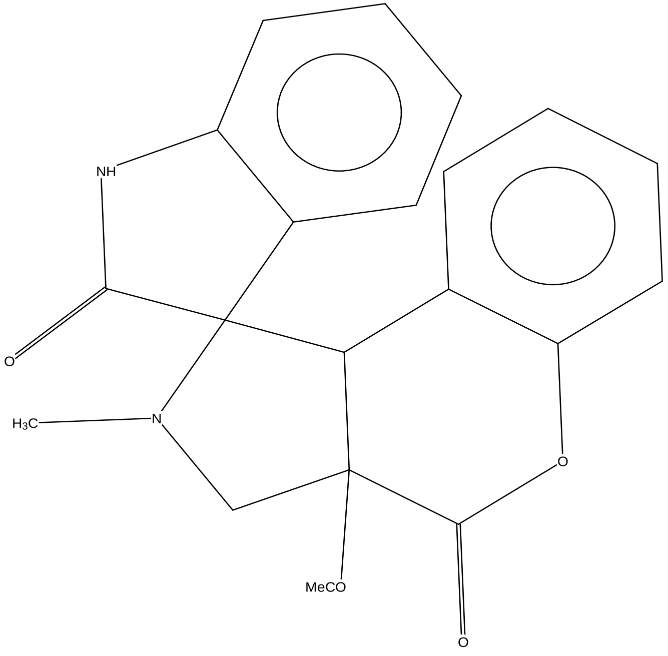

Parameters

Fragment 1

ANG1 (A) 87.655

SUTLEZ

Reference:	M.Ghandi, A.Taheri, A.Abbasi (2010) *Tetrahedron* ,**66,** 6744
Formula:	C_{19} H_{15} Br_1 N_2 O_3
Compound Name:	8-Bromo-2-methyl-2,3,3a,9b-tetrahydro-4H-spiro[chromeno[3,4-c]pyrrole-1,3'-indole]-2',4(1'H)-dione

Space Group:	P21/c	***Cell:***	***a*** 10.859(2)	***b*** 13.440(3)	***c*** 11.917(2)
Space Group No.:	14	***(Å,°)***	α 90.00	β 105.14(3)	γ 90.00
R-Factor (%):	6.51	***Temperature(K):*** 295		***Density(g/cm^3):*** 1.580	

Parameters

Fragment 1
ANG1 (A) 86.597

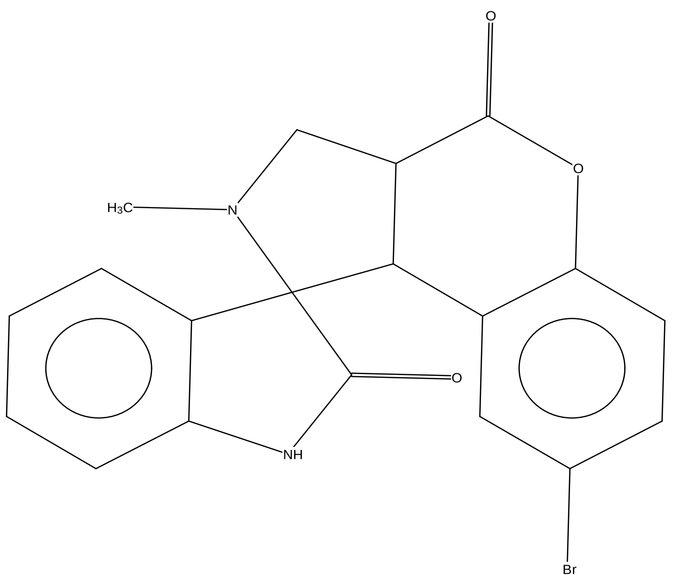

Supplement: Supplementary file 4 [file e-75-00246-sup4.pdf]
