# Supplementary material for: Crystal structures of 6a,6b,7,11a-tetra­hydro-6H,9H-spiro­[chromeno[3′,4′:3,4]pyrrolo­[1,2-c]thia­zole-11,3′-indoline]-2′,6-dione and 5′-methyl-6a,6b,7,11a-tetra­hydro-6H,9H-spiro­[chromeno[3′,4′:3,4]pyrrolo­[1,2-c]thia­zole-11,3′-indoline]-2′,6-dione
Source: Acta Crystallogr E Crystallogr Commun. 2019 Jan 22;75(Pt 2):246–50. doi: 10.1107/S2056989019000045 (PMC6362654; doi:10.1107/S2056989019000045)

Search Overview

Search:	search4
Date/Time done:	Sat Dec 29 17:01:03 2018
Database(s):	CSD version 5.39 updates (Nov 2017)<br>CSD version 5.39 (November 2017)<br>CSD version 5.39 (November 2017)<br>CSD version 5.39 updates (Feb 2018)<br>CSD version 5.39 updates (May 2018)<br>CSD version 5.39 updates (Aug 2018)
Restriction Info:	No refcode restrictions applied
Filters:	None
Percentage Completed:	100%
Number of Hits:	23

Single query used. Search found structures that:

match

Query 2

Query 2

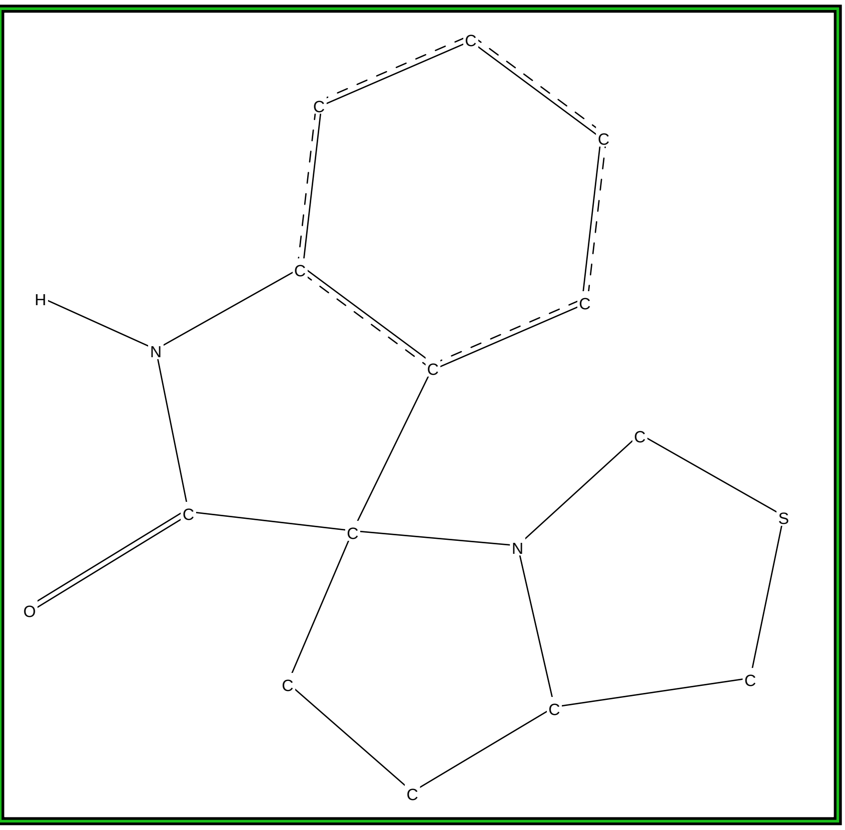

Search: search4 (Sat Dec 29 17:01:03 2018): Hits 1-2

AJIKEL

Reference: C.Mhiri, S.Boudriga, M.Askri, M.Knorr, D.Sriram, P.Yogeeswari, F.Nana, C.Golz, C.Strohmann (2015) *Bioorg.Med.Chem.Lett.* ,**25,**4308

Formula: $C_{26}\ H_{18}\ Br_2\ N_2\ O_3\ S_1$

Compound Name: 5''-bromo-7'-(4-bromophenyl)-7',7a'-dihydro-1'H-dispiro[1-benzofuran-3, 6'-pyrrolo[1,2-c][1,3]thiazole-5',3''-indole]-2,2''(1''H)-dione

Space Group:	P-1	***Cell:***	***a*** 8.831(0)	***b*** 11.282(0)	***c*** 13.473(0)
Space Group No.:	2	***(Å,°)***	α 68.87(0)	β 72.34(0)	γ 88.47(0)
R-Factor (%):	3.40	***Temperature(K):*** 173		***Density(g/cm³):*** 1.673	

Parameters

Fragment 1
ANG1 (A) 88.242

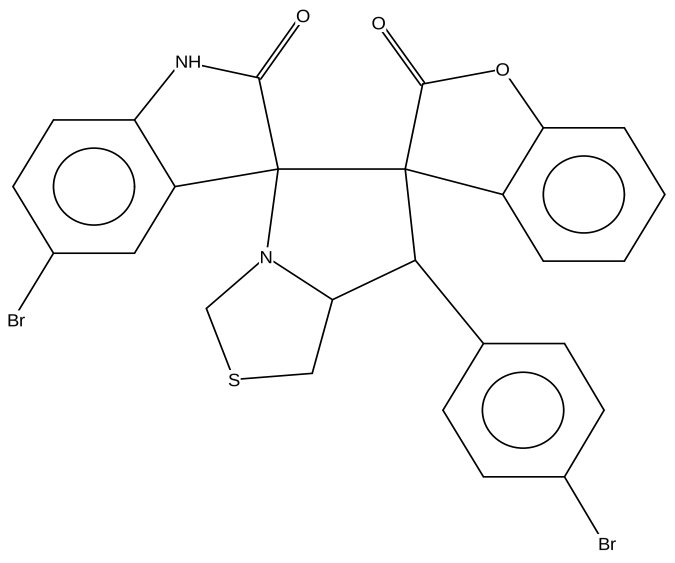

ALAREL

Reference: S.U.Maheswari, K.Balamurugan, S.Perumal, P.Yogeeswari, D.Sriram (2010) *Bioorg.Med.Chem.Lett.* ,**20,**7278

Formula: $C_{28}\ H_{22}\ N_2\ O_4\ S_1$

Compound Name: 7'-(4-Methoxyphenyl)-7',7a'-dihydro-1'H-dispiro[indene-2,6'-pyrrolo[1,2-c][1,3]thiazole-5',3''-indole]-1,2'',3(1''H)-trione

Space Group:	P21/c	***Cell:***	***a*** 9.596(1)	***b*** 10.279(1)	***c*** 24.202(4)
Space Group No.:	14	***(Å,°)***	α 90.00	β 93.00(1)	γ 90.00
R-Factor (%):	5.29	***Temperature(K):*** 293		***Density(g/cm³):*** 1.345	

Parameters

Fragment 1
ANG1 (A) 80.655

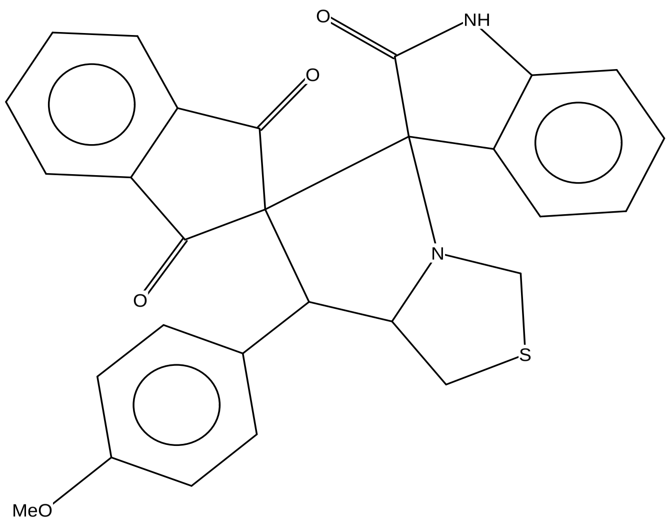

ALARIP

Reference:	S.U.Maheswari, K.Balamurugan, S.Perumal, P.Yogeeswari, D.Sriram (2010) *Bioorg.Med.Chem.Lett.* ,**20,**7278
Formula:	C_{27} H_{19} Cl_1 N_2 O_3 S_1
Compound Name:	7'-(4-Chlorophenyl)-7',7a'-dihydro-1'H-dispiro[indene-2,6'-pyrrolo[1,2-c][1,3]thiazole-5',3''-indole]-1,2'',3(1''H)-trione

Space Group:	P21/c	***Cell:***	***a***	9.691(1)	***b***	10.491(1)	***c***	22.510(3)
Space Group No.:	14	***(Å,°)***	α	90.00	β	93.46(1)	γ	90.00
R-Factor (%):	5.84	***Temperature(K):***	293		***Density(g/cm³):***	1.416		

Parameters

Fragment 1
ANG1 (A) 81.939

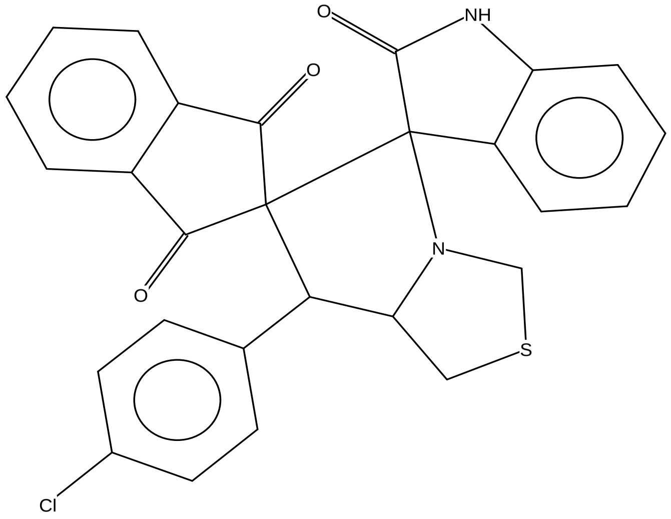

BUGRAX

Reference:	S.V.Karthikeyan, B.D.Bala, V.P.A.Raja, S.Perumal, P.Yogeeswari, D.Sriram (2010) *Bioorg.Med.Chem.Lett.* ,**20,**350
Formula:	C_{33} H_{33} N_3 O_2 S_1
Compound Name:	1''-Methyl-5''-(4-methylbenzylidene)-7'-(4-methylphenyl)-7',7a'-dihydro-1'H,4''H-dispiro[indole-3,5'-pyrrolo[1,2-c][1,3]thiazole-6',3''-piperidine]-2,4''(1H)-dione

Space Group:	C2/c	***Cell:***	***a***	13.736	***b***	13.038	***c***	31.967
Space Group No.:	15	***(Å,°)***	α	90.00	β	97.82	γ	90.00
R-Factor (%):	4.60	***Temperature(K):***	293		***Density(g/cm³):***	1.255		

Parameters

Fragment 1
ANG1 (A) 84.430

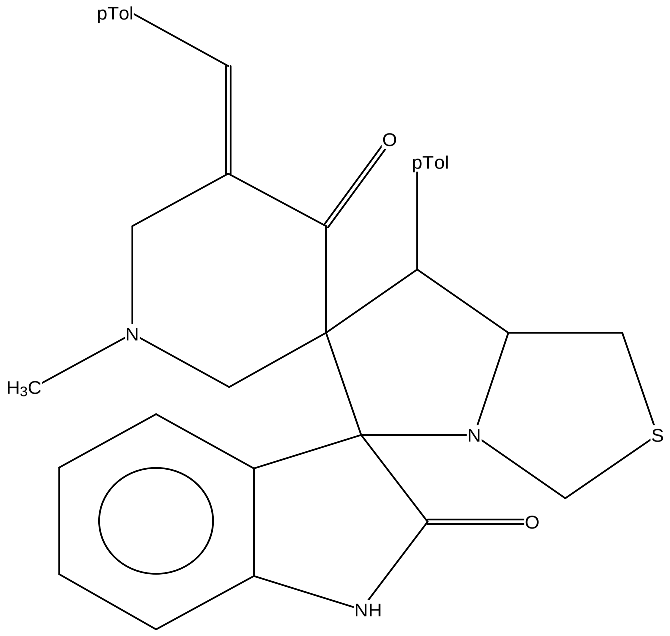

BUGREB

Reference:	S.V.Karthikeyan, B.D.Bala, V.P.A.Raja, S.Perumal, P.Yogeeswari, D.Sriram (2010) *Bioorg.Med.Chem.Lett.* ,**20,**350
Formula:	C_{31} H_{27} Cl_2 N_3 O_2 S_1,H_2 O_1
Compound Name:	5''-(4-Chlorobenzylidene)-7'-(4-chlorophenyl)-1''-methyl-7',7a'-dihydro-1'H,4''H-dispiro[indole-3,5'-pyrrolo[1,2-c][1,3]thiazole-6',3''-piperidine]-2,4''(1H)-dione monohydrate

Space Group:	P21/c	***Cell:***	***a***	11.136(5)	***b***	16.186(5)	***c***	16.566(5)
Space Group No.:	14	***(Å,°)***	α	90.00	β	95.14(0)	γ	90.00
R-Factor (%):	5.67	***Temperature(K):***	293		***Density(g/cm³):***	1.328		

Parameters

Fragment 1
ANG1 (A) 86.125

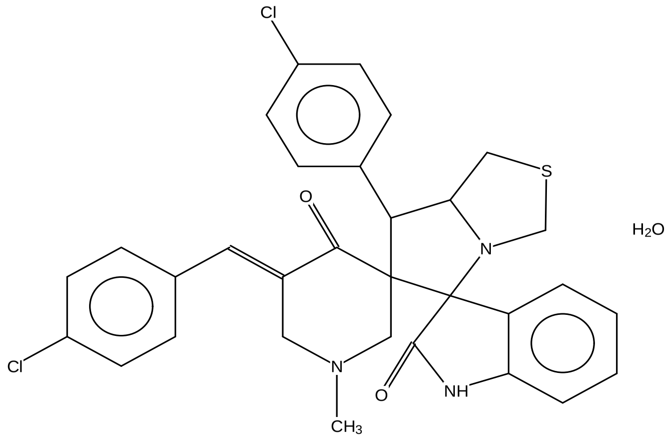

EMOKIC

Reference:	R.S.Kumar, A.I.Almansour, N.Arumugam, S.M.Soliman, R.R.Kumar, H.A.Ghabbour (2016) *J.Mol.Struct.* ,**1121,**93
Formula:	C_{35} H_{35} N_3 O_4 S_1
Compound Name:	1''-allyl-5''-(4-methoxybenzylidene)-7'-(4-methoxyphenyl)-7',7a'-dihydro-1'H,4''H-dispiro[indole-3,5'-pyrrolo[1,2-c][1,3]thiazole-6',3''-piperidine]-2,4''(1H)-dione

Space Group:	P21/c	***Cell:***	***a***	11.618(0)	***b***	12.247(0)	***c***	21.706(0)
Space Group No.:	14	***(Å,°)***	α	90.00	β	103.02(0)	γ	90.00
R-Factor (%):	5.27	***Temperature(K):***	100		***Density(g/cm³):***	1.311		

Parameters

Fragment 1
ANG1 (A) 82.528

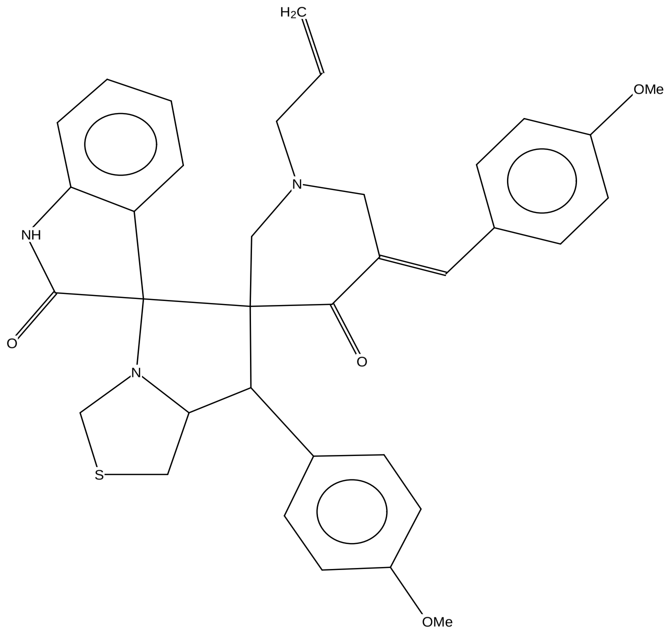

EMONEA

Reference:	P.Prasanna, K.Balamurugan, S.Perumal, P.Yogeeswari, D.Sriram (2010) *Eur.J.Med.Chem.* ,**45,**5653
Formula:	$C_{27}\ H_{21}\ Cl_1\ N_2\ O_2\ S_1$
Compound Name:	7'-(2-Chlorophenyl)-7',7a'-dihydro-1'H-dispiro[indene-2,6'-pyrrolo[1,2-c][1,3]thiazole-5',3''-indole]-1,2''(1''H,3H)-dione

Space Group:	Pbca	***Cell:***	***a*** 17.148(1)	***b*** 14.681(0)	***c*** 18.777(1)		
Space Group No.:	61	***(Å,°)***	α 90.00	β 90.00	γ 90.00		
R-Factor (%):	3.78	***Temperature(K):***	273	***Density(g/cm³):***	1.329		

Parameters

Fragment 1
ANG1 (A) 87.232

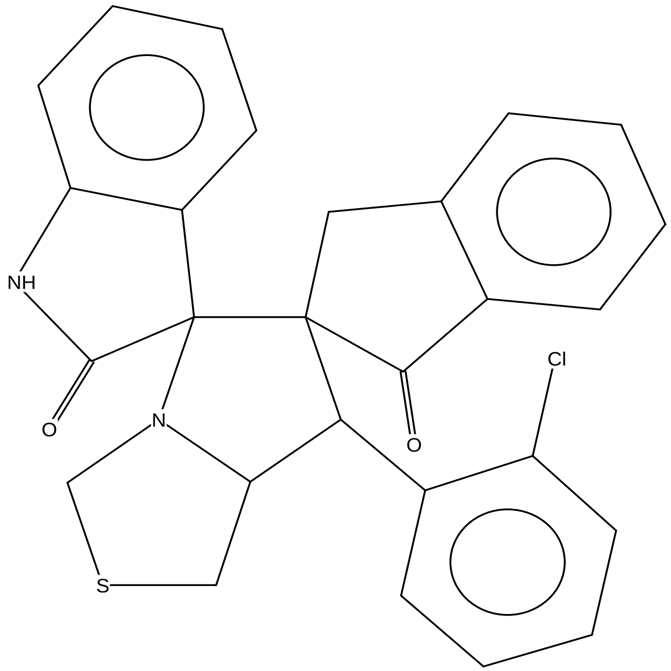

FOGXUV

Reference:	S.Selvanayagam, J.Paul, D.Velmurugan, K.Ravikumar, R.R.Durga, R.Raghunathan (2005) *Acta Crystallogr.,Sect.E:Struct.Rep.Online* ,**61,**o1585
Formula:	$C_{28}\ H_{24}\ N_2\ O_4\ S_1$
Compound Name:	7'-(4-Methoxyphenyl)-chroman-3-spiro-6'-hexahydro-1H-pyrrolo(1,2 -c) thiazole- 5'-spiro-3''-1''H-indole-4,2''(3''H)-dione

Space Group:	P21/c	***Cell:***	***a*** 9.556(0)	***b*** 16.104(1)	***c*** 15.263(1)		
Space Group No.:	14	***(Å,°)***	α 90.00	β 91.21(1)	γ 90.00		
R-Factor (%):	4.70	***Temperature(K):***	273	***Density(g/cm³):***	1.371		

Parameters

Fragment 1
ANG1 (A) 84.068

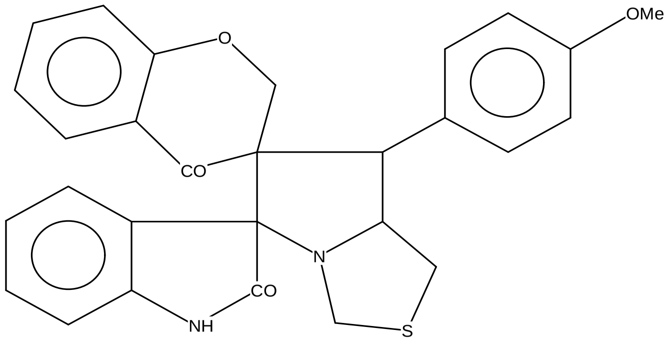

Search: search4 (Sat Dec 29 17:01:03 2018): Hits 9-10

FOWLAG

Reference: S.Haddad, S.Boudriga, F.Porzio, A.Soldera, M.Askri, D.Sriram, P.Yogeeswari, M.Knorr, Y.Rousselin, M.M.Kubicki (2014) *RSC Advances* ,**4,**59462

Formula: C_{28} H_{23} N_3 O_3 S_1

Compound Name: 1'',7'-diphenyl-7',7a'-dihydro-1'H,2''H,5''H-dispiro[indole-3,5'-pyrrolo[1,2-c][1,3]thiazole-6',3''-pyrrolidine]-2,2'',5''(1H)-trione

Space Group:	P-1	***Cell:***	***a*** 9.137(0)	***b*** 10.062(1)	***c*** 14.303(1)		
Space Group No.:	2	***(Å,°)***	α 94.06(0)	β 99.91(0)	γ 114.79(0)		
R-Factor (%):	3.36	***Temperature(K):***	115	***Density(g/cm³):***	1.378		

Parameters

Fragment 1
ANG1 (A) 78.746

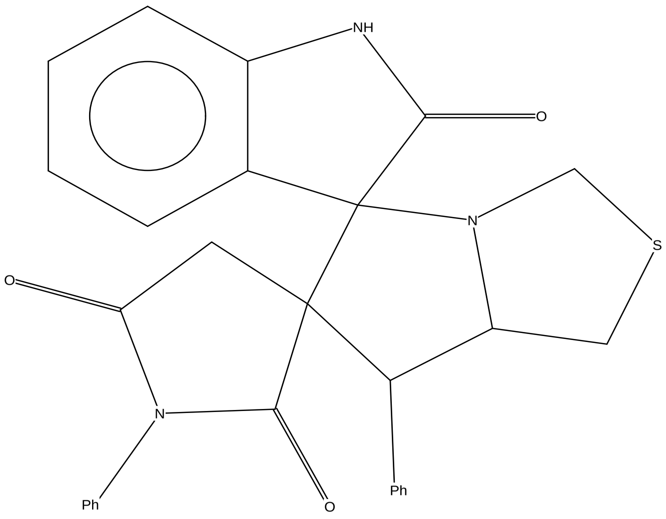

GITDOD

Reference: S.Sundaramoorthy, D.Gayathri, D.Velmurugan, M.Poornachandran, K.Ravikumar (2008) *Acta Crystallogr.,Sect.E:Struct.Rep.Online* ,**64,**o488

Formula: C_{25} H_{26} N_2 O_2 S_1

Compound Name: 1'-Phenyl-6'-thiacycloheptane-1-spiro-2'-perhydropyrrolizine-3'-spiro-3''-indoline-2,2''-dione

Space Group:	P-1	***Cell:***	***a*** 8.985(1)	***b*** 10.356(1)	***c*** 12.812(1)
Space Group No.:	2	***(Å,°)***	α 80.15(0)	β 71.01(0)	γ 67.50(0)
R-Factor (%):	6.23	***Temperature(K):***	293	***Density(g/cm³):***	1.337

Parameters

Fragment 1
ANG1 (A) 77.600

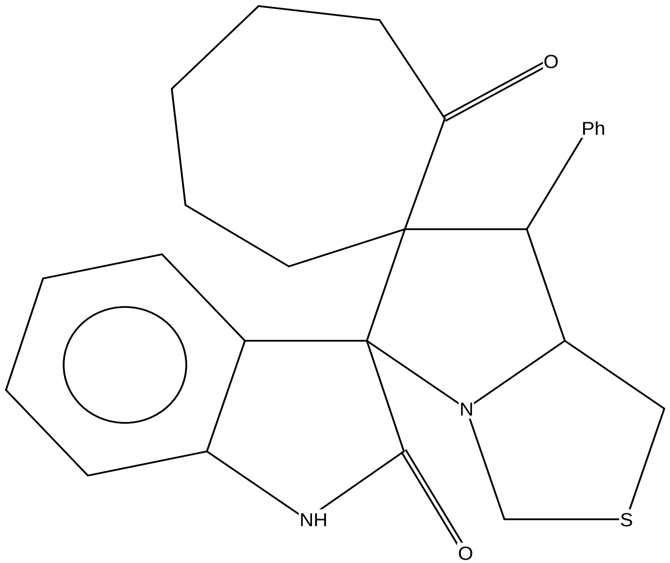

GUCHET

Reference: Jie Li, Jing Wang, Zhou Xu, Songlei Zhu (2014) *ACS Comb. Sci.* ,**16,**506

Formula: $C_{25}\ H_{20}\ Cl_1\ N_3\ O_2\ S_1,C_2\ H_6\ O_1$

Compound Name: 7'-(2-Chlorophenyl)-6'-(pyridin-2-ylcarbonyl)-1',6',7',7a'-tetrahydrospiro[indole-3,5'-pyrrolo[1,2-c][1,3]thiazol]-2(1H)-one ethanol solvate

Space Group:	P21/n	***Cell:***	***a***	11.552(0)	***b***	16.654(0)	***c***	13.774(0)
Space Group No.:	14	***(Å,°)***	α	90.00	β	106.45(0)	γ	90.00

R-Factor (%): 7.74 ***Temperature(K):*** 293 ***Density(g/cm³):*** 1.328

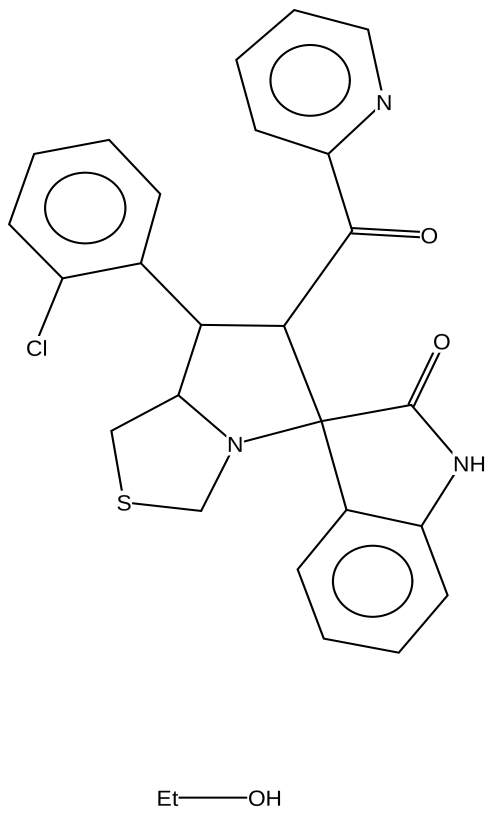

Parameters

Fragment 1
ANG1 (A) 79.942

JAWTOT

Reference: Mani Anusha Rani, S.V.Kumar, K.Malathi, M.Muthu, A.I.Almansour, R.S.Kumar, R.R.Kumar (2017) *ACS Comb. Sci.* ,**19,**308

Formula: $C_{29}\ H_{22}\ Cl_2\ N_2\ O_2\ S_2$

Compound Name: (Z)-5''-(2-Chlorobenzylidene)-7'-(2-chlorophenyl)-7',7a'-dihydro-1'H,2''H,3'H-dispiro[indoline-3,5'-pyrrolo[1,2-c]thiazole-6',3''-thiophene]-2,4'' (5''H)-dione

Space Group:	P21/c	***Cell:***	***a***	10.409(0)	***b***	11.041(0)	***c***	23.459(1)
Space Group No.:	14	***(Å,°)***	α	90.00	β	93.02(0)	γ	90.00

R-Factor (%): 5.14 ***Temperature(K):*** 293 ***Density(g/cm³):*** 1.395

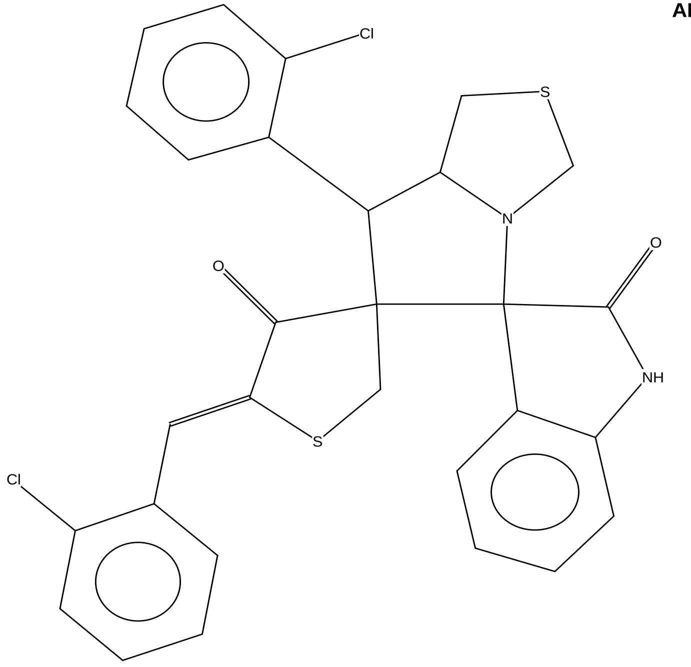

Parameters

Fragment 1
ANG1 (A) 87.247

MUDLAA

Reference: S.Kanchithalaivan, Mani Anusha Rani, R.R.Kumar (2014) *Synth.Commun.* ,**44,**3122

Formula: $C_{38}\ H_{46}\ N_2\ O_3\ S_1$

Compound Name: 3-hydroxy-10,13-dimethyl-7'-(4-methylphenyl)-1,3,4,5,6,7,7',7a',8,9,10,11,12,13,14,15-hexadecahydro-1'H-dispiro[cyclopenta[a]phenanthrene-16,6'-pyrrolo[1,2-c][1,3]thiazole-5',3''-indole]-2'',17(1''H,2H)-dione

Synonym: (16R,5'R,7'R,7a'R)-spiro[5'.3'']oxindole-spiro[6'.16]-7-(4-methylphenyl)-tetrahydro-1H-pyrrolo[1,2-c][1,3]thiazolo-trans-dehydroandrosterone

**Space Group:**	C2	**Cell:**	**a** 22.689(0)	**b** 12.825(0)	**c** 11.431(0)
**Space Group No.:**	5	**(Å,°)**	α 90.00	β 99.73(0)	γ 90.00

R-Factor (%): 4.99 **Temperature(K):** 293 **Density(g/cm³):** 1.238

Parameters

Fragment 1
ANG1 (A) 89.712

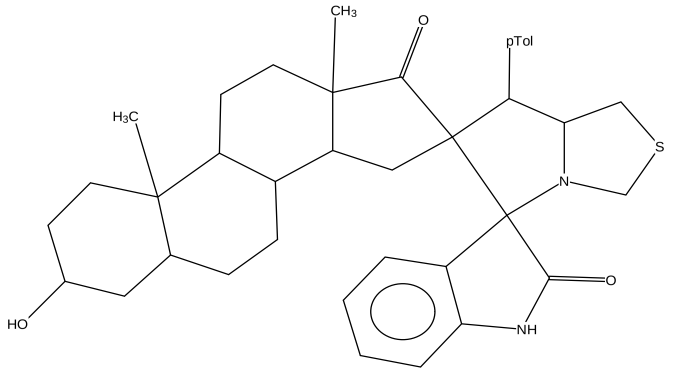

NUHHIJ

Reference: S.Suhitha, T.Srinivasan, R.Prasanna, K.Gunasekaran, R.Raghunathan, D.Velmurugan (2013) *Int.J.ChemTech.Res.* ,**5,**2793

Formula: $C_{30}\ H_{34}\ N_2\ O_7\ S_1$

Compound Name: ethyl 7'-(6-(benzyloxy)-2,2-dimethyltetrahydrofuro[2,3-d][1,3]dioxol-5-yl)-2-oxo-1,1',2,6',7',7a'-hexahydrospiro[indole-3,5'-pyrrolo[1,2-c][1,3]thiazole]-6'-carboxylate

**Space Group:**	P212121	**Cell:**	**a** 9.378(0)	**b** 12.622(0)	**c** 23.992(1)
**Space Group No.:**	19	**(Å,°)**	α 90.00	β 90.00	γ 90.00

R-Factor (%): 4.56 **Temperature(K):** 293 **Density(g/cm³):** 1.325

Parameters

Fragment 1
ANG1 (A) 87.785

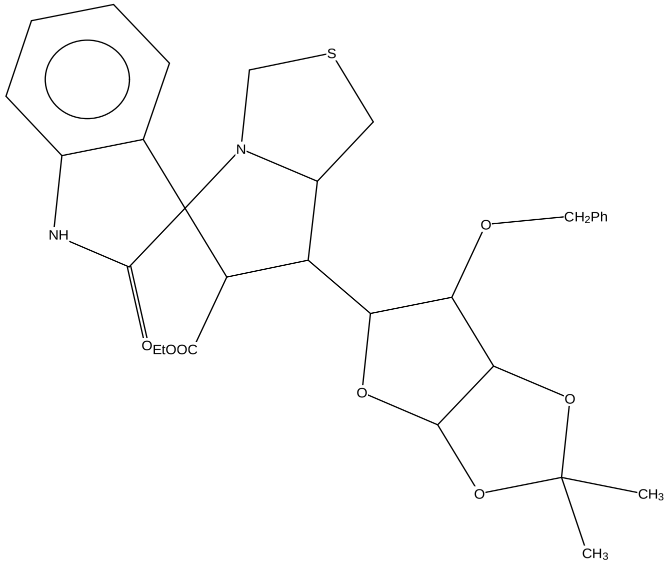

QEXMEM

Reference:	D.Gayathri, S.Kalyani, D.Velmurugan, K.Ravikumar, M.Poornachandran (2007) *Acta Crystallogr.,Sect.E:Struct.Rep.Online* , **63,**o852
Formula:	$C_{26} H_{28} N_2 O_2 S_1$
Compound Name:	7'-Phenyl-cyclooctane-1-spiro-6'-2-thiapyrrolizidine-5'-spiro-3''-indole-2, 2''(3H)-dione

Space Group:	P-1	***Cell:***	***a*** 9.090(0)	***b*** 10.501(0)	***c*** 12.751(0)
Space Group No.:	2	***(Å,°)***	α 79.87(0)	β 71.50(0)	γ 68.57(0)
R-Factor (%):	4.65	***Temperature(K):***	293	***Density(g/cm³):***	1.340

Parameters

Fragment 1
ANG1 (A) 78.252

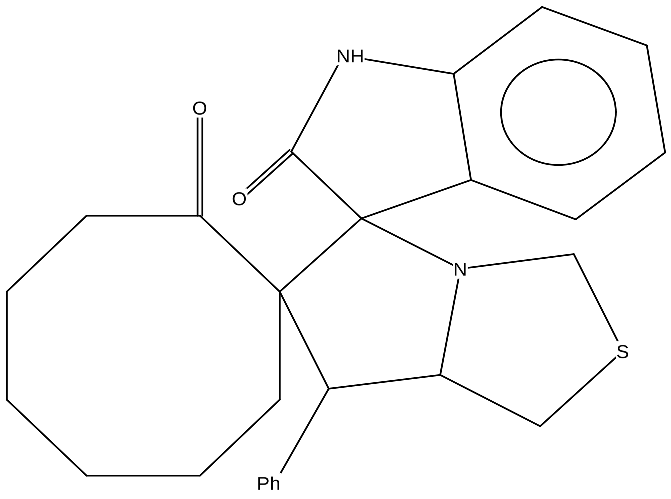

QUBVEQ

Reference:	M.P.Savithri, M.Suresh, R.Raghunathan, R.Raja, A.SubbiahPandi (2015) *Acta Crystallogr.,Sect.E:Cryst.Commun.* ,**71,** o148
Formula:	$C_{23} H_{20} N_2 O_4 S_2$
Compound Name:	Ethyl 2'',3-dioxo-1'',2'',7',7a'-tetrahydro-1'H,3H-dispiro[1-benzothiophene-2,6'-pyrrolo[1,2-c][1,3]thiazole-5',3''-indole]-7'-carboxylate
Synonym:	Ethyl 2'',3-dioxo-7',7a'-dihydro-1'H,3H,3'H-dispiro[benzo[b]thiophene-2, 6'-pyrrolo[1,2-c]thiazole-5',3''-indoline]-7'-carboxylate

Space Group:	P21/c	***Cell:***	***a*** 11.889(0)	***b*** 10.218(0)	***c*** 17.504(0)
Space Group No.:	14	***(Å,°)***	α 90.00	β 97.99(0)	γ 90.00
R-Factor (%):	3.19	***Temperature(K):***	293	***Density(g/cm³):***	1.427

Parameters

Fragment 1
ANG1 (A) 81.985

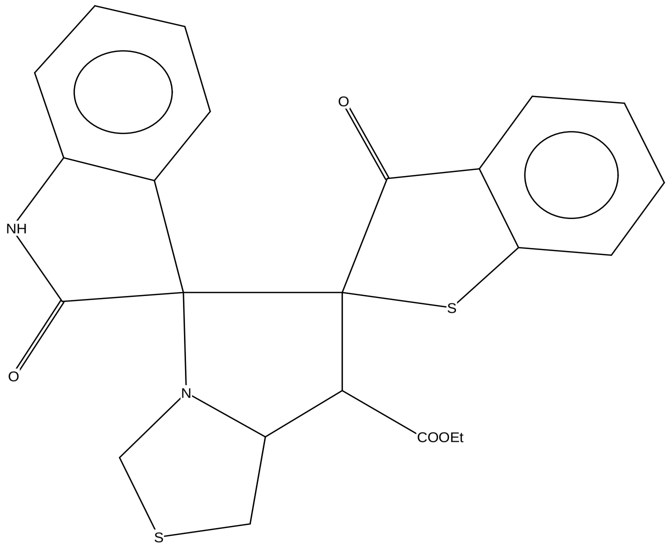

RAGMUK

Reference: G.Lotfy, M.M.Said, El S.H El Ashry, El S.H El Tamany, A.Al-Dhfyan, Y.M.A.Aziz, A.Barakat (2017) *Bioorg.Med.Chem.* ,**25,**1514

Formula: C_{31} H_{28} N_2 O_2 S_1

Compound Name: (1R,5'R)-3-benzylidene-7'-phenyl-7',7a'-dihydro-1'H,2H-dispiro[cyclohexane-1,6'-pyrrolo[1,2-c][1,3]thiazole-5',3''-indole]-2,2''(1''H)-dione

Space Group:	P-1	***Cell:***	***a*** 7.772(0)	***b*** 10.508(0)	***c*** 15.496(0)
Space Group No.:	2	***(Å,°)***	α 97.10(0)	β 100.91(0)	γ 97.84(0)

R-Factor (%): 5.59 ***Temperature(K):*** 100 ***Density(g/cm³):*** 1.345

Parameters

Fragment 1
ANG1 (A) 83.636

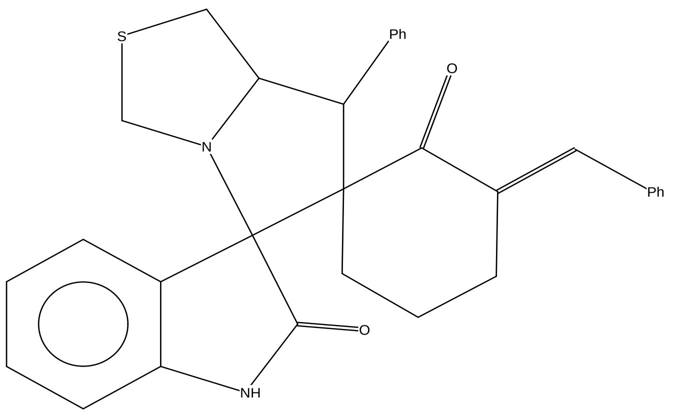

RAGNAR

Reference: G.Lotfy, M.M.Said, El S.H El Ashry, El S.H El Tamany, A.Al-Dhfyan, Y.M.A.Aziz, A.Barakat (2017) *Bioorg.Med.Chem.* ,**25,**1514

Formula: C_{33} H_{32} N_2 O_2 S_1

Compound Name: (1R,5'R)-3-(3-methylbenzylidene)-7'-(3-methylphenyl)-7',7a'-dihydro-1'H,2H-dispiro[cyclohexane-1,6'-pyrrolo[1,2-c][1,3]thiazole-5',3''-indole]-2,2''(1''H)-dione

Space Group:	P-1	***Cell:***	***a*** 7.662(1)	***b*** 10.634(3)	***c*** 16.593(4)
Space Group No.:	2	***(Å,°)***	α 93.93(0)	β 98.07(0)	γ 91.40(0)

R-Factor (%): 10.79 ***Temperature(K):*** 100 ***Density(g/cm³):*** 1.296

Parameters

Fragment 1
ANG1 (A) 82.989

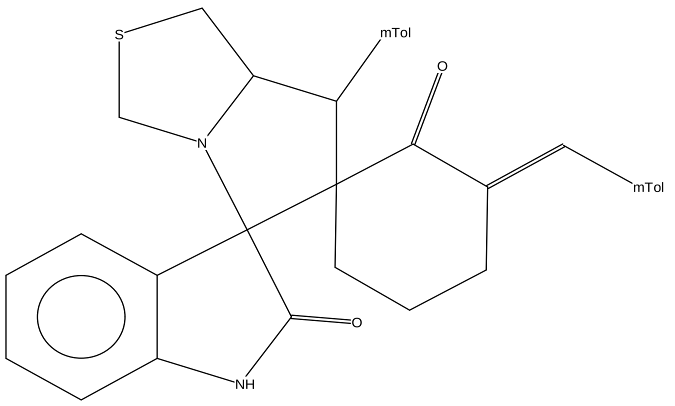

RAHBIO

Reference:	G.Lotfy, M.M.Said, El S.H El Ashry, El S.H El Tamany, A.Al-Dhfyan, Y.M.A.Aziz, A.Barakat (2017) *Bioorg.Med.Chem.* ,**25,**1514
Formula:	C_{27} H_{24} N_2 O_2 S_3
Compound Name:	(1R,5'R,E)-7'-(2-thienyl)-3-(2-thienylmethylene)-7',7a'-dihydro-1'H,2H-dispiro[cyclohexane-1,6'-pyrrolo[1,2-c][1,3]thiazole-5',3''-indole]-2,2''(1''H)-dione

Space Group:	P-1	***Cell:***	***a*** 7.828(1)	***b*** 10.445(1)	***c*** 15.031(2)
Space Group No.:	2	***(Å,°)***	α 96.63(0)	β 100.78(0)	γ 99.81(0)
R-Factor (%):	8.27	***Temperature(K):*** 100		***Density(g/cm^3):*** 1.425	

Parameters

Fragment 1
ANG1 (A) 83.594

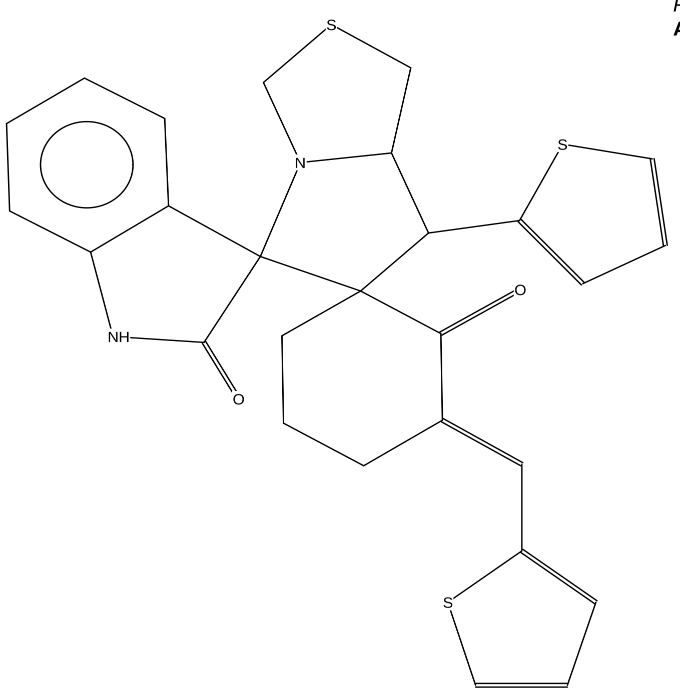

SUWNEE

Reference:	R.Prasanna, S.Purushothaman, R.Raghunathan (2010) *Tetrahedron Lett.* ,**51,**4538
Formula:	C_{27} H_{34} N_2 O_8 S_1,H_2 O_1
Compound Name:	Ethyl 2-oxo-7'-(2,2,7,7-tetramethyltetrahydro-3aH-bis[1,3]dioxolo[4,5-b:4',5'-d]pyran-5-yl)-1,1',2,6',7',7a'-hexahydrospiro[indole-3,5'-pyrrolo[1,2-c][1,3]thiazole]-6'-carboxylate monohydrate
Synonym:	1,2:3,4-diisopropylidene-5-C[Spiro[2'.3'']oxindolo-3'-ethoxycarbonyl-thiapyrrolizidine]-D-galactopyranose monohydrate

Space Group:	P212121	***Cell:***	***a*** 9.098(2)	***b*** 9.853(3)	***c*** 30.855(8)
Space Group No.:	19	***(Å,°)***	α 90.00	β 90.00	γ 90.00
R-Factor (%):	6.38	***Temperature(K):*** 293		***Density(g/cm^3):*** 1.356	

Parameters

Fragment 1
ANG1 (A) 84.475

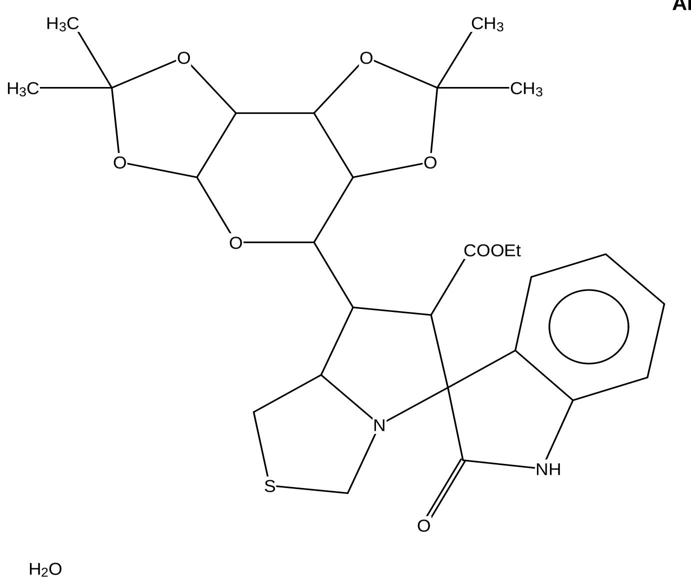

TEJRUW

Reference: A.Subbiahpandi, D.Velmurugan, K.Ravikumar, E.Ramesh, R.Raghunathan (2006) *Acta Crystallogr.,Sect.E:Struct.Rep.Online* ,**62,** o2259

Formula: $C_{37}\ H_{32}\ N_2\ O_2\ S_1$

Compound Name: 3-Benzylidene-3',7'-diphenylcyclohexanespiro-6'-(perhydro-2-thiapyrrolizine)-5'-spiro-3''-(1H-indole)-2,2''-dione

Space Group:	P21/c	***Cell:***	***a*** 10.031(0)	***b*** 20.860(1)	***c*** 14.131(0)
Space Group No.:	14	***(Å,°)***	α 90.00	β 100.97(0)	γ 90.00
R-Factor (%):	5.14	***Temperature(K):***	293	***Density(g/cm³):***	1.301

Parameters

Fragment 1
ANG1 (A) 81.015

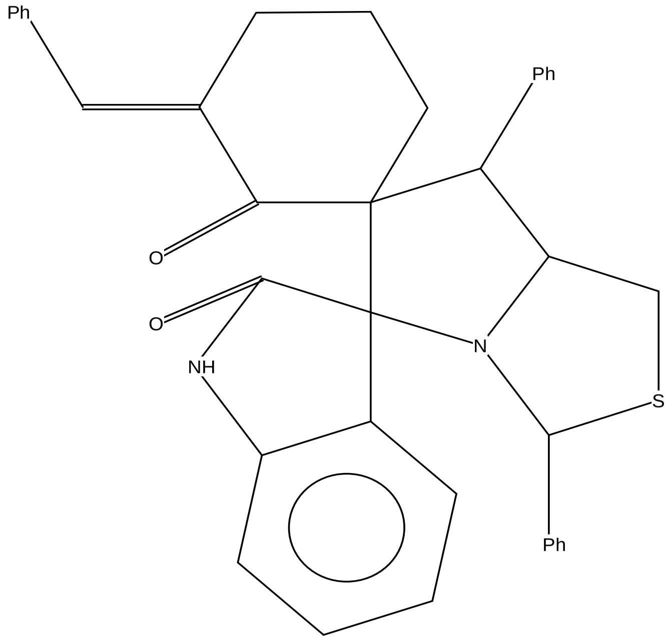

XEVGIQ

Reference: A.Kumar, G.Gupta, S.Srivastava, A.K.Bishnoi, R.Saxena, R.Kant, R.S.Khanna, P.R.Maulik, A.Dwivedi (2013) *RSC Advances* ,**3,** 4731

Formula: $C_{32}\ H_{25}\ Cl_1\ N_2\ O_2\ S_1$

Compound Name: 6'-Benzoyl-7'-(4-chlorophenyl)-3'-phenyl-1',6',7',7a'-tetrahydrospiro[indole-3,5'-pyrrolo[1,2-c][1,3]thiazol]-2(1H)-one

Space Group:	P21/c	***Cell:***	***a*** 10.428(5)	***b*** 16.883(5)	***c*** 17.610(5)
Space Group No.:	14	***(Å,°)***	α 90.00	β 119.48(1)	γ 90.00
R-Factor (%):	7.04	***Temperature(K):***	293	***Density(g/cm³):***	1.322

Parameters

Fragment 1
ANG1 (A) 81.438

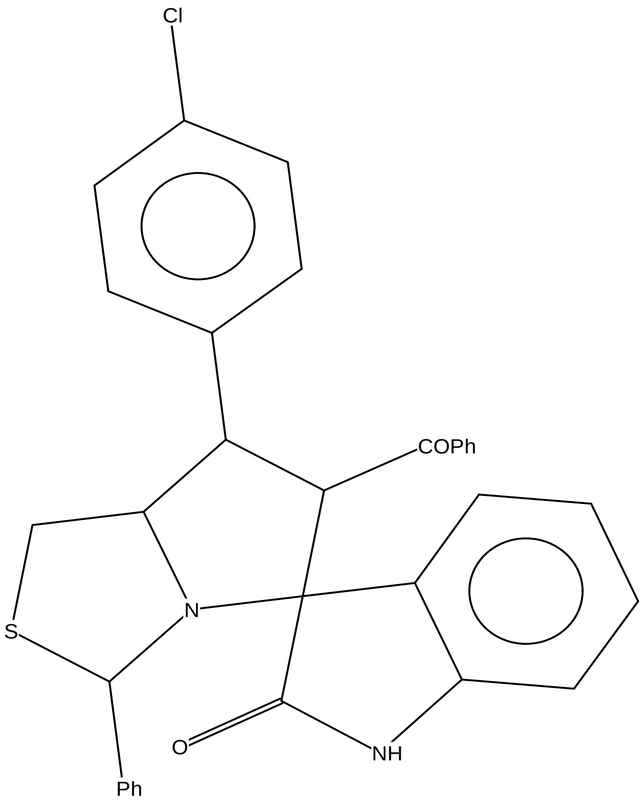

Search: search4 (Sat Dec 29 17:01:03 2018): Hit 23

XIJQUE

Reference:	A.A.Shvets, Yu.V.Nelyubina, K.A.Lyssenko, S.V.Kurbatov<br>(2012) *Izv.Akad.Nauk SSSR,Ser.Khim.(Russ.)(Russ.Chem.Bull.)* ,1642						
Formula:	$C_{25} H_{20} N_4 O_3 S_3$						
Compound Name:	3''-(2-Furylmethyl)-7'-(pyridin-3-yl)-2''-thioxo-7',7a'-dihydro-1'H,4''H-dispiro[indole-3,5'-pyrrolo[1,2-c][1,3]thiazole-6',5''-[1,3]thiazolidine]-2,4''(1H)-dione						
Space Group:	P21/c	***Cell:***	***a***	12.960(0)	***b*** 17.871(1)	***c***	10.060(0)
Space Group No.:	14	***(Å,°)***	α	90.00	β 95.04(0)	γ	90.00
R-Factor (%):	4.84	***Temperature(K):***	120		***Density(g/cm³):*** 1.490		

Parameters

Fragment 1

ANG1 (A) 88.254

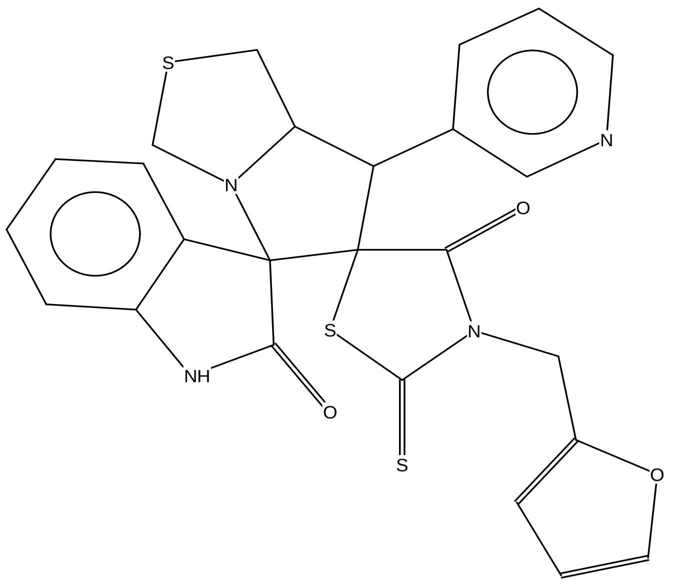

Supplement: Supplementary file 5 [file e-75-00246-sup5.pdf]
